# Scalable photonic network architecture based on motional averaging in room temperature gas

**DOI:** 10.1038/ncomms11356

**Published:** 2016-04-14

**Authors:** J. Borregaard, M. Zugenmaier, J. M. Petersen, H. Shen, G. Vasilakis, K. Jensen, E. S. Polzik, A. S. Sørensen

**Affiliations:** 1 The Niels Bohr Institute, University of Copenhagen, Blegdamsvej 17, Copenhagen Ø DK-2100, Denmark; 2 Department of Physics, Harvard University, Cambridge, Massachusetts 02138, USA

## Abstract

Quantum interfaces between photons and atomic ensembles have emerged as powerful tools for quantum technologies. Efficient storage and retrieval of single photons requires long-lived collective atomic states, which is typically achieved with immobilized atoms. Thermal atomic vapours, which present a simple and scalable resource, have only been used for continuous variable processing or for discrete variable processing on short timescales where atomic motion is negligible. Here we develop a theory based on motional averaging to enable room temperature discrete variable quantum memories and coherent single-photon sources. We demonstrate the feasibility of this approach to scalable quantum memories with a proof-of-principle experiment with room temperature atoms contained in microcells with spin-protecting coating, placed inside an optical cavity. The experimental conditions correspond to a few photons per pulse and a long coherence time of the forward scattered photons is demonstrated, which is the essential feature of the motional averaging.

## Abstract (web summary)

Cold atomic ensembles have been considered suitable platforms to realize quantum memories, but their scalability is limited by the cooling apparatuses. Here, the authors show how room temperature atomic microcells can be used for discrete variable ensemble-based quantum information processing.

## Graphical abstract

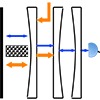

Quantum systems can potentially enable powerful quantum computation^1^,^2^,^3^ and highly secure quantum networks^4^,^5^,^6^,^7^. Especially for the latter, it is essential that the information can be stored in quantum memories for processing^6^,^8^. To this end, ensembles of cold atoms have previously been considered for quantum memories since their large number of atoms enables a strong light–atom interaction^9^,^10^,^11^,^12^. Cold atoms, however, require an extended cooling apparatus, which makes the scalability of such systems challenging. In contrast, room temperature atoms are much simpler to work with and have been used for a range of operations with continuous variables^13^. Several experiments with room temperature atomic cells have exploited a form of motional averaging where atoms move in and out of the beam many times during the interaction. By using a spin-preserving coating of the walls of the cell^13^, the atoms can return back into the beam after colliding with a cell wall without losing the phase information. The averaging thereby removes the detrimental effect of atomic motion for continuous variable quantum information processing, where a specific optical mode is measured by homodyne detection^14^,^15^,^16^. On the other hand, for discrete variables protocols based on the detection of photon clicks, the situation is different since photon counters select an almost instantaneous temporal mode. As a consequence, the efficiency of such systems for discrete variables where, for example, a single-spin excitation is stored collectively in the ensemble, is still limited by the incoherent atomic motion, which leaks which atom information and collapses the collective state^17^.

Here, we introduce room temperature microcells as a system for discrete variable, ensemble-based quantum information processing. We show theoretically how the detrimental effect of atomic motion can be circumvented in order to have an efficient and coherent interaction between an atomic ensemble and light at the single-photon level. The proposed technique can be used to make efficient single-photon sources with memories and offers a solution to scalable photonic networks based on room temperature atoms. The spin-protecting microcells investigated here can also be used for long distance quantum communication in DLCZ-like repeater protocols^6^,^7^,^18^ or quantum simulations^19^,^20^,^21^. The scalability of the system compared with cryogenic or cold atoms systems opens up the possibility of employing large arrays of such systems combined with spatial multiplexing to enhance the communication rate^8^,^22^. As opposed to previous ensemble-based experiments with discrete variables encoded in moving atoms^23^, which typically rely on performing operations sufficiently fast that the atoms remain inside the laser beams, we show how a form of motional averaging similar to the one used for continuous variable processing can be used to erase the which atom information. This approach to photon-counting experiments alleviates the effect of atomic motion and can be seen as ‘trapping' coherent spins in a box-like potential made up of the coated walls. We also present a proof-of-principle experiment demonstrating the effect. Besides the specific experimental realization described here, the ideas behind this form of motional averaging are generally applicable and may be used in other systems where fluctuations of the coupling strength is an issue, such as ion crystals^24^. A related motional averaging of frequency fluctuations has previously been considered in super-conducting qubits^25^.

## Results

### Setup

**Figure 1:**
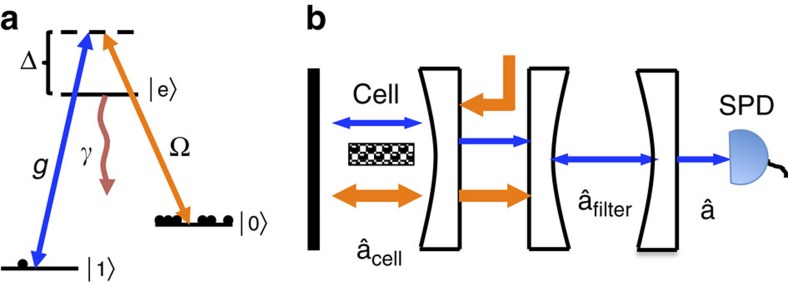
Atomic level structure and experimental setup. (**a**) All atoms are initially pumped to state |0〉. The transition |0〉→|*e*〉 is driven by a weak laser field (Ω), while the cavity mode (*g*) couples |*e*〉 and |1〉. The driving is far detuned 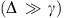 from the excited level to suppress the effects of Doppler broadening and absorption. *γ* is the decay rate of the excited level |*e*〉. (**b**) The atomic ensemble is kept in a small cell inside a single-sided cavity with a low finesse (cell cavity). The quantum photons (thin arrows) are coupled from the cell cavity into a high-finesse cavity (filter cavity), which separates them from the classical field (thick arrows) and averages over the atomic motion. Finally, the quantum photons are measured with a SPD. We associate the quantum field inside the cell cavity (filter cavity) with an annihilation operator 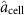 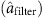, while the field at the detector is associated with the annihilation operator 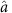. SPD, single-photon detector.

We consider a setup where an ensemble of atoms with a Λ-scheme level structure is kept in a small alkene-coated cell^26^,^27^ (see Fig. 1). A cell with quadratic cross section with side length 2*L*=300 μm containing caesium atoms was used in ref. 28, for which the average time between atom–wall collisions was ∼1.4 μs and the coherence time was 10 ms. The atoms can thus endure several collisions with the walls before losing coherence making the cells suitable as quantum memories. The ensemble is kept at room temperature and, to enhance the interaction with the light, the cell is placed inside a single-sided optical cavity (‘cell' cavity). In the proof-of-principle experiment (see below) a finesse of 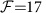 has been set by the output mirror transmission of 20% and the reflection losses on the cell windows but a cavity with a higher finesse 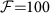 can easily be envisioned. The light leaving the cell cavity is coupled into another high-finesse cavity (‘filter' cavity), whose purpose is described below.

**Figure 2:**
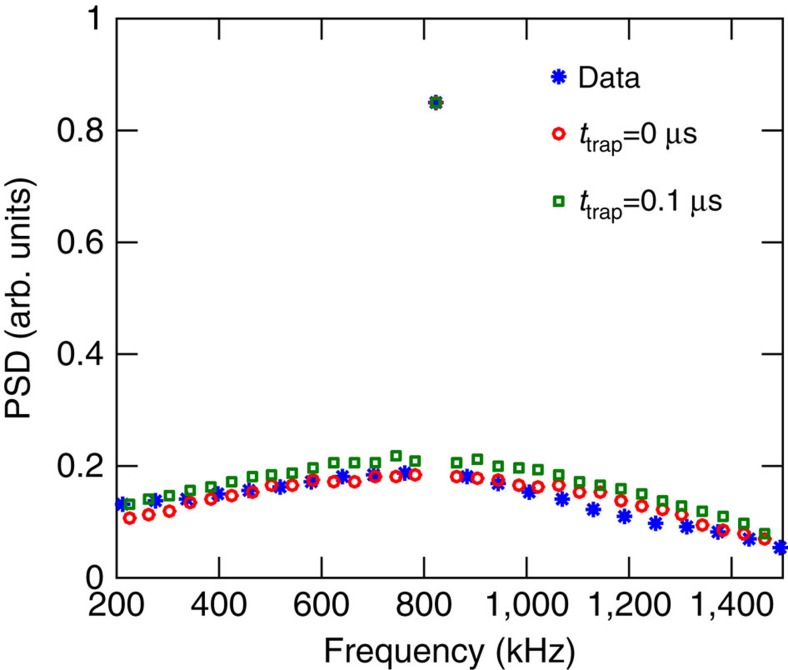
PSD of the emitted light. The figure shows both the experimental data and the simulation of the PSD. The broad feature originates from short-time, incoherent light–atom interaction, while the sharp peak is from the long-time, coherent light–atom interaction. The optical scattering was obtained through the Faraday effect and is centred around the Larmor frequency at 823.8 kHz (the single high point in the figure). The simulations have been rescaled to coincide with the data at this point. In the simulations, we include the possibility of atoms being trapped in the coating of the cell walls. From the figure, we estimate that such trapping time is below 0.1 μs and can thus be ignored. The cell used in the experiment had dimensions 2*L* × 2*L* × 2*L*_*z*_ with *L*=150 μm and *L*_*z*_=0.5 cm and the light beam had a Gaussian profile with a waist of 55 μm. The statistical uncertainty of each experimental point is very small and is therefore not shown.

Initially, all atoms are pumped to a stable ground state |0〉 (see Fig. 1). In the ‘write' process, the objective is to create a single, collective excitation in the ensemble, thereby creating the symmetric Dicke state 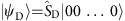, with 
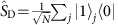
 where *j* is the atom number, *N* is the total number of atoms and |1〉 is another stable ground state in the atoms. The |0〉→|*e*〉 transition is driven with a laser pulse, which is far-detuned from the atomic transition to suppress the effect of the Doppler broadening of the atomic levels and absorption. In addition, the pulse should be sufficiently weak such that multiple excitations in the ensemble can be neglected. The write process is conditioned on detecting a single photon (quantum photon) emitted in a Raman transition |0〉→|*e*〉→|1〉. Upon detection, the atomic state is projected into the symmetric Dicke state if the light experienced a homogeneous interaction with all atoms, that is, if the probability for different atoms to have emitted the photon is equal. In a realistic setup, the laser beam does not fill the entire cell and only atoms that are in the beam contribute to the cavity field, resulting in an asymmetric spin wave being created. Atoms leaving the beam will, however, return to the beam due to the frequent collisions with the cell walls. During the collisions, the atomic state is preserved because of the alkene coating of the cells and we exploit this to make a motional averaging of the light–atom interaction. If the interaction time is long enough to allow the atoms to move in and out of the beam several times, they will on average have experienced the same interaction with the light. Consequently, the detection of a cavity photon will, to a good approximation, project the atomic state to a Dicke state. Since the cell cavity has a limited finesse, it may, in practice, not have a sufficiently narrow linewidth to allow this averaging. We therefore introduce an external filter cavity. As we show below, the output from the cell cavity consists of a spectrally narrow coherent component and a broad incoherent component (see Fig. 2). By selecting out the coherent part, the filter cavity effectively increases the interaction time and allows for motional averaging. At the same time, the filter cavity can also separate the quantum photon from the classical drive if there is a small frequency difference between the two, such that only one frequency is resonant in the filter cavity while both are sustained in the cell cavity. Furthermore, choosing the frequency difference to be an even number of free spectral ranges of the cell cavity ensures an overlap of the field modes at the centre of the cavity, such that atoms can interact simultaneously with both modes if the length of the microcell is small compared with the wavelength corresponding to the frequency difference (see Supplementary Methods and Supplementary Fig. 1).

After a successful creation of an excitation in the ensemble, the state can be kept until it is read out. In the readout process, a long classical pulse addresses the |1〉→|*e*〉 transition, such that the single excitation is converted into a photon on the |*e*〉→|0〉 transition (Fig. 1a with *g* and Ω interchanged). This pulse should be long enough to allow for motional averaging as in the write process. The filter cavity can once again be used to filter the quantum photon from the classical drive photons. Furthermore, it can also be used to filter away incoherent photons as described below.

### Write process

To characterize the quality of our system, when considered as a single-photon source with memory, we first derive the efficiency of the write process and later discuss the readout efficiency and quality of the single photons being retrieved. The Hamiltonian, describing the write process, is (*ħ*=1)


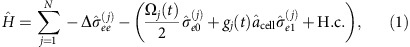


where Δ=*ω*_laser_−*ω*_*e*0_ with *ω*_laser_ being the frequency of the driving laser and *ω*_*e*0_ being the transition frequency between the levels |*e*〉 and |0〉. We have assumed that the cavity is on resonance with photons emitted on the |*e*〉→|1〉 transition (see Fig. 1a). Ω_*j*_ (*g*_*j*_) characterizes the coupling between the laser (cavity) field and the *j*'th atom. The field in the cell cavity is described by the annihilation operator 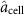 and we have defined the atomic operators 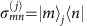 for the *j*'th atom, where {*m*, *n*}∈{0, 1, *e*}. To obtain an expression for the cavity field, we formally integrate Heisenberg's equations of motion including the cavity (*κ*_1_) and atomic (*γ*) decays. The field at the detector (see Fig. 1b), described by 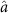, is found by propagating 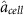 through the filter cavity. Treating the interaction as a perturbation to the atomic system and omitting noise operators, we find (see Methods section)


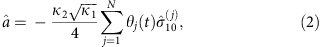


where


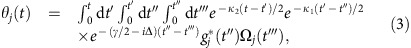


with *κ*_2_ being the decay rate of the filter cavity.

The efficiency is defined as the probability of having stored a single excitation in the symmetric Dicke state upon detection of a quantum photon. Neglecting higher order excitations, the atomic state is projected to 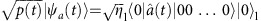 when the quantum photon is detected at time *t*. Here, |0〉_l_ is the vacuum of the light in the cavity mode and *p*(*t*) is the probability density of detecting the photon at time *t* with *η* being the single-photon detection efficiency. Assuming that the driving pulse has a duration of *t*_int_, the efficiency of the write process is


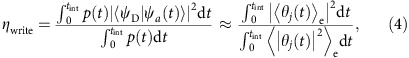


where we have used equation (2), assumed 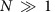, and have defined the ensemble average 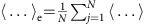.

To get an expression for *η*_write_, we need to evaluate 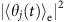 and 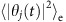 which is done in detail in the Methods section. Here, it is important to note that while 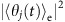 does not contain any correlations between an atom's position at different times, 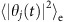 does. Equation (4) thus characterizes the effect of the random atomic motion and the motional averaging associated with it. The correlations decay in time such that after many collisions with the walls, an atom's position is completely uncorrelated from its initial position. To evaluate the correlations, we perform Monte Carlo simulations of individual atoms in a rectangular cell moving in and out of the cavity beam and experiencing random collisions with the walls. Evaluating the correlation including the Doppler shift, we find that the decay of the correlations are approximately exponential such that, for example, 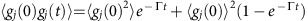, where the first term contains the short-time correlations, while the second term characterizes the long-time limit where the correlations are only through the average values. Employing this model for the atomic correlations and assuming the effective interaction time (1/*κ*_2_) is set by the linewidth of the filter cavity, we find


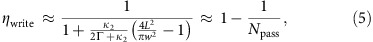


where *w* is the width of the Gaussian beam profile of the cavity fields and 2*L* is the transverse size of the cell. We have assumed *L*>*w*, 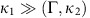 and that we are detuned beyond the Doppler width of the atomic levels. Equation (5) shows that *η*_write_→1 as *κ*_2_/Γ→0, that is, the write efficiency improves with the length of the effective interaction time. This is the motional averaging of the atomic interaction with the light. Equation (5) also shows how the efficiency improves as the ratio between the beam area and the cell area (*πw*^2^/*L*^2^) increases. The last equality in equation (5) is obtained assuming that Γ>>*κ*_2_. In this limit 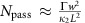 can be interpreted as the average number of passes of an atom through the beam during the decay time of the filter cavity.

**Figure 3:**
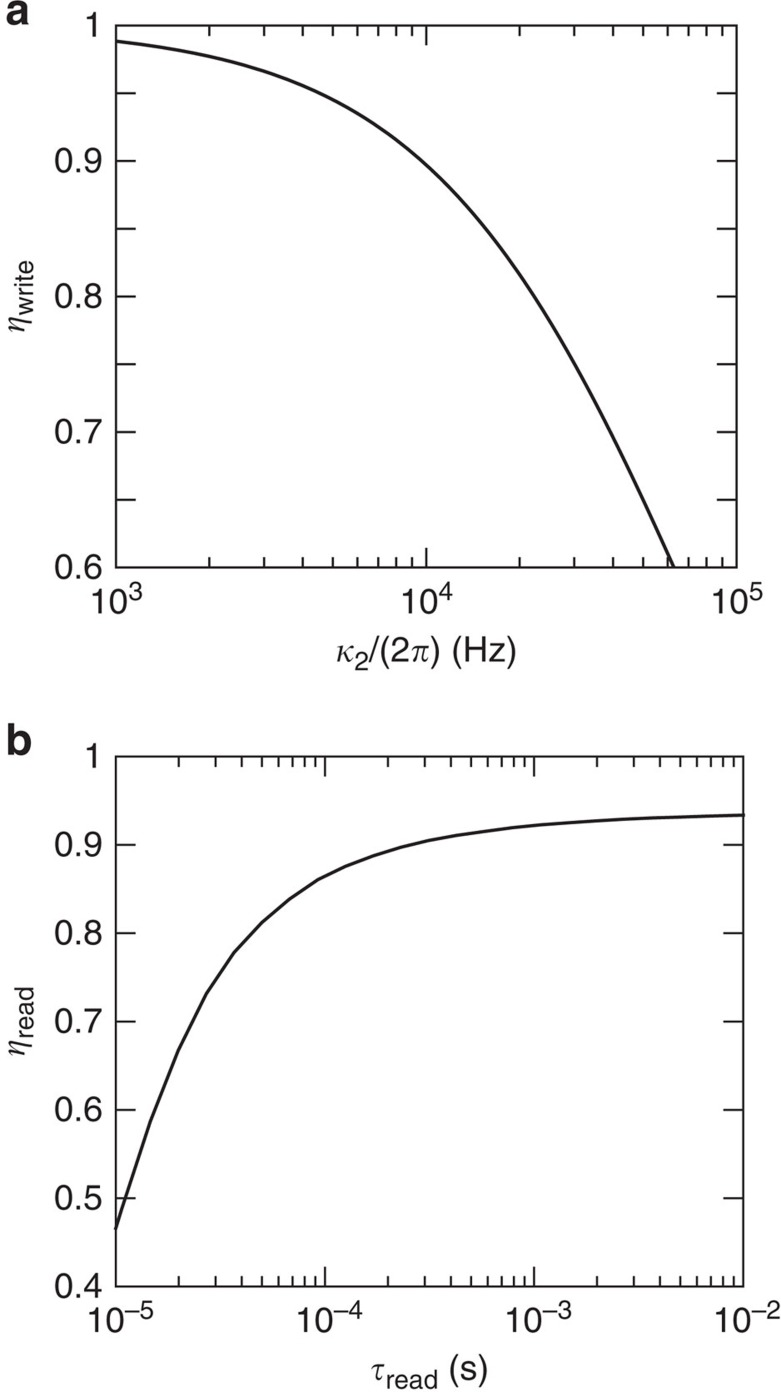
Write and read efficiency. (**a**) Write efficiency as a function of the linewidth of the filter cavity *κ*_2_. We have simulated a Cs-cell with wall length 2*L*=300 μm and cavity beam waist *w*=55 μm corresponding to the cells being used in the proof-of-principle experiment. We have assumed a detuning of Δ∼2*π*·900 MHz, a pulse length of *t*_int_=10/*κ*_2_ and a cell-cavity decay rate *κ*_1_=2*π*·46 MHz. (**b**) Optimal readout efficiency as a function of the readout time *τ*_read_ without the filter cavity (corresponding to *κ*_2_→∞). The efficiency was simulated for the same Cs-cells as the write efficiency and we have assumed that 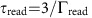 where Γ_read_ is the readout rate, which is proportional to the classical drive intensity. The optical depth was assumed to be 168 as measured in the experiment. The finesse of the filter cavity was varied between 20 and 100 to get the optimal readout efficiency. We have included the full-level structure of ^133^Cs in the simulations (see Supplementary Methods and Supplementary Fig. 4).

To describe the write efficiency quantitatively, we have numerically simulated the experiment with Cs-cells including the full-level structure of the atoms as described in Supplementary Methods. The Λ-scheme level structure can be realized with the two ground states |0〉=|*F*=4, *m*_*F*_=4〉 and |1〉=|*F*=3, *m*_*F*_=3〉 in the 6^2^*S*_1/2_ ground-state manifold and the excited state |*e*〉=|*F*′=4, *m*_*F*′_=4〉 in the excited 6^2^*P*_3/2_ manifold. Note that with this configuration, the quantum and classical field differ both in polarization and frequency and the filtering of the quantum photon is thus expected to be easily obtained using a combination of both polarization filtering and the filter cavity. Figure 3a shows the simulated write efficiency as a function of *κ*_2_. It is seen that *η*_write_≈90% for *κ*_2_≈2*π*·10 kHz, which translates into a write time of ∼160 μs. Furthermore, we estimate that the number of classical photons, which should be filtered from the quantum photon is ∼4.4 × 10^5^ for realistic experimental parameters (see Supplementary Methods). This level of filtering is expected to be easily achieved using frequency filtering.

### Proof-of-principle experiment

To confirm the validity of the model and the results obtained above, we have performed a proof-of-principle experiment, which confirms the most important prediction, the presence of a spectrally narrow coherent peak of the scattered light arising from motional averaging. While several previous experiments^14^,^15^,^16^,^26^ have demonstrated long coherence times of room temperature atoms, we wish to show a long coherence time of the emitted photons, thus demonstrating that the motional averaging technique can be exploited to make coherent photon emission. To do this, we compare the theoretical predictions with the experimentally observed power spectral density (PSD) of light scattered by the atoms. In this proof-of-principle experiment, linearly polarized probe light, off-resonant from the atomic transition, interacts with the atoms resulting in Faraday paramagnetic rotation of the light polarization^13^ and the polarization state of the light is recorded with balanced polarimetry. As explained below, the balanced polarimetry establishes a heterodyne measurement of the Raman scattered photons allowing us to determine their spectrum.

**Figure 4:**
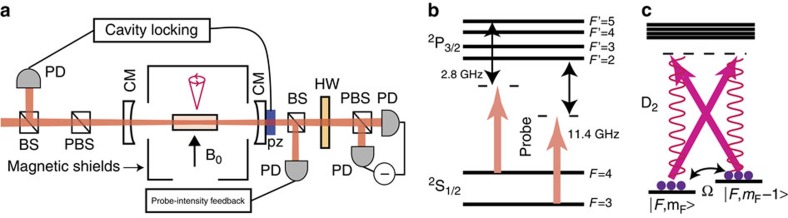
Proof-of-principle experiment. (**a**) Schematic representation of the proof-of-principle experiment. (**b**) The D2 transition probed in the proof-of-principle experiment. (**c**) Effective coupling scheme for the Faraday interaction^13^. The strong probe beam (straight arrows) is polarized perpendicular to the applied field and can thus drive *σ*_+_ and *σ*_−_ transition. An atom scattered between two different *m*_*F*_ levels results in a *π* polarized photon (wiggly lines), orthogonal to the drive. In the weak probing limit, the measurement of the Faraday rotation angle is thus equivalent to a heterodyne measurement of the emitted light in the Raman transition with the probe pulse acting as a local oscillator. BS, beam splitter; CM, cavity mirror; PBS, polarizing beam splitter; PD, photodiode.

The experimental setup is shown in Fig. 4 and is further explained in the Methods section. A DC bias magnetic field perpendicular to the probe direction sets the Larmor frequency of the atoms. Because of technical limitations related with the phase noise of the laser and the cell birefringence, the polarization of the probe was at an angle of ∼40–45° with respect to the axis of the magnetic field. When the probe light is far detuned, the Faraday rotation is, however, independent of this angle^13^. For simplicity, we therefore describe the dynamics using the level structure in Fig. 4c, which assumes that the driving field is *σ*_+_+*σ*_−_ polarized, perpendicular to the direction of the magnetic field *π*. In the far-detuned limit, the Faraday rotation is due to Raman transitions between magnetic states with magnetic quantum numbers *m*_*F*_ differing by ±1. In these Raman transitions, a *π*-polarized photon is emitted as shown in Fig. 4c. In the balanced polarimetry, the driving field and the scattered *π* component of the light are mixed on a polarizing beam splitter and the difference intensity is recorded. This corresponds to the driving field acting as local oscillator for a heterodyne measurement of the emitted π-polarized light. The recorded Raman noise is thus a measurement of the photons emitted from the atoms through Raman scattering between the Zeeman sublevels of the Cs hyperfine manifolds and is therefore exactly the quantity we are interested in for probing the coherence of the emitted photons and verifying the predictions of the model. The experiment is performed in the continuous regime with constant laser intensity. By comparing the emitted light to the shot-noise level, we can extract the Raman scattering rate. For a pulse duration that can lead to an efficient write step, for example, 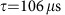, corresponding to *κ*_2_=2*π*·15 kHz in Fig. 3a, we find that approximately eight Raman photons are scattered in the upper sideband mode (see Supplementary Methods and Supplementary Fig. 2). Because of the linearity of the process, the spectrum is expected to be the same at the single-photon level.

The measured PSD and the simulations of it are shown in Fig. 2. The measured Raman noise reflects two different correlation decay timescales: a fast decay timescale ∼1 μs associated with the transient time of flight through the probe beam; and a relatively slow decay ∼100 μs, due to the spin decoherence (probe induced spin relaxation). Since the spectrum shown in Fig. 2 is measured for the scattered light, it is seen that the long-spin coherence translates into a long coherence time of photons at the single-photon level consistent with the theory. The PSD is recorded with a higher frequency resolution than shown in the figure but we bin the data with a frequency resolution (Δ*f*≈61 kHz) chosen, so that the Raman noise of atoms in the two hyperfine manifolds associated with the slow correlation decay timescale is contained in a single frequency bin. By doing this, complications arising from the nonlinear Zeeman splitting and the difference in the gyromagnetic ratio between the different hyperfine manifolds can be ignored. The simulations were carried out as described in Supplementary Methods and have been rescaled to fit the measured single-photon detector at 823.7 kHz. We have carried out simulations both where the atomic collisions with the wall coatings happen instantaneously (*t*_trap_=0), that is, so that the trapping time is negligible compared with the transient time, and with a trapping time of *t*_trap_=0.1 μs. Figure 2 shows an excellent agreement between the experiment and the model with zero trapping time. From Fig. 2, we estimate that any trapping time in the experiment is 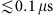 and can thus be ignored. The narrow peak in the scattered light is due to the fact that atoms repeatedly come back into the beam with the same spin phase, whereas the broad background is due to single transients through the beam. The narrow peak in the data thus demonstrates the long coherence time of the forward scattered light due to motional averaging. Considering the random motion of the atoms, the only coherence that can survive for that long is linked to the symmetric Dicke state as described in the theory. In essence, the idea of the motional averaging is to use a spectrally narrow filter cavity to select only the photons emitted in the narrow coherent peak. Since this narrow peak corresponds to a long interaction time, this means that all atoms participate equally in the resulting spin wave. Furthermore, since the narrow peak is much higher than the broad background, the loss in efficiency from the spectral filtering is limited. The excellent agreement between the simulation and the experiment thus confirms the applicability of the motional averaging as well as the theoretical model we use.

### Readout

We now consider the readout process. Assuming a single excitation has been stored in the symmetric mode in the ensemble, a classical drive (Ω) is applied to read out the excitation as a cavity photon. The relevant Hamiltonian is obtained by interchanging 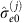 and 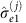 in equation (1). From Heisenberg's equations of motion, we obtain a set of *N*+1 coupled differential equations of the cavity field 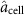 and the atomic operators 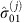 (see Methods section). The equations can be expressed as a matrix system of the form


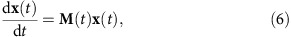


where 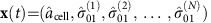 and **M**(*t*) is the coupling matrix between the atoms and the cavity field. The coupling matrix can be expressed as **M**(*t*)=**M**_0_+*δ***M**(*t*) where **M**_0_ contains the average time-independent couplings, while *δ***M**(*t*) contains the time-dependent fluctuations. Assuming the readout pulse is long, the atoms will have had the same average interaction with the light meaning that **M**(*t*)≈**M**_0_. Treating *δ***M**(*t*) as a small perturbation, we can then obtain a perturbative expansion of 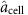. Assuming that the initial state of the atoms before readout is the symmetric Dicke state, we find that, to second order in *δ***M**(*t*), the cavity field can be expressed as 
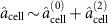
. Here we have omitted the first-order term since we find that it is suppressed by a factor of at least 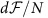 compared with the other terms. Here 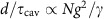 is the optical depth on the |0〉↔|*e*〉 transition per cavity roundtrip time and 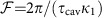 is the finesse of the cell cavity.

As in the write process, the field from the cell cavity is sent through the filter cavity in order to both filter the classical drive photons from the single photon and to filter out incoherent photons as we will describe below. As described in the Methods section, we find that the readout efficiency is


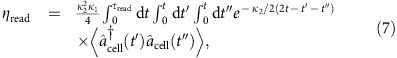


where 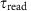 is the duration of the readout pulse. To lowest order we find a zeroth-order readout efficiency of


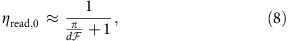


in the limit of a very long and weak readout pulse. Equation (8) is equivalent to the result for cold atomic ensembles^29^ and represents the long-time limit of perfect motional averaging, where the efficiency improves with optical depth and finesse of the system.

The coherence time of real atoms is, however, limited and a fast readout is therefore desirable. The readout rate Γ_read_ increases with increasing strength of the readout pulse (see Methods section), so that for strong driving corresponding to a fast readout, it is necessary to consider higher order terms in the perturbative expansion of 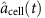. To second order, we find that 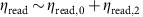 where the second-order term (*η*_read,2_) mainly describes the loss of the excitation due to spontaneous emission. Consequently, the magnitude of *η*_read,2_ increases with the driving strength while its sign is negative. *η*_read,2_ contains correlations between an atom's position at different times, which we can treat in a similar manner as in the write process, that is, as exponentially decaying in time. By simulating the readout process with the Cs-cells in a similar fashion as for the write process, we can quantitatively describe the readout efficiency to second order (see Supplementary Methods). Figure 3b shows the readout efficiency to second order as a function of the readout time. We have assumed an optical depth of 168 and varied the finesse of the cell cavity between 20–100 to get the maximum readout efficiency. The readout time is set to 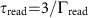 ensuring that a negligible population is left in the system at the end of the readout stage. (Note that in these simulations, we do not include the filter cavity considered for the write stage. Formally, this corresponds to taking the limit *κ*_2_→∞.) The full-level structure of ^133^Cs is included in the simulations and the optimization in the finesse is due to the extra levels in Cs-atoms, which introduces additional couplings. In general, high (low) finesse is optimal for short (long) readout times. A small cavity detuning was also included in the optimization in order to compensate for the shifts caused by the couplings to the extra levels (see Supplementary Methods). For a finesse of ∼50 and a readout time of *t*_read_≈200 μs, a readout efficiency of *η*_read_≈90% is obtained.

### Errors

So far, we have focused on the efficiency of the protocol. We will now consider the errors, which limit the performance of the system as a single-photon source with memory. We find that the dominant errors are multiple excitations during the write process and the possibility of reading out atoms, which have been incoherently moved to state |1〉 by either inefficient optical pumping or wall collisions.

Multiple excitations in the write process would also create multiple quantum photons, which could in principle be discriminated from the situation with a single quantum photon if perfect single-photon detection is possible. In a realistic setup there will, however, always be some finite detection probability *η*_d_ and the probability of creating two excitations would introduce an error of 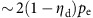 to lowest order where 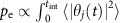 is the excitation probability. This error can be made arbitrarily small by simply decreasing *p*_e_, that is, decreasing the strength of the classical drive. This will, however, also decrease the rate of the operation, which scales as 1/*p*_e_.

**Figure 5:**
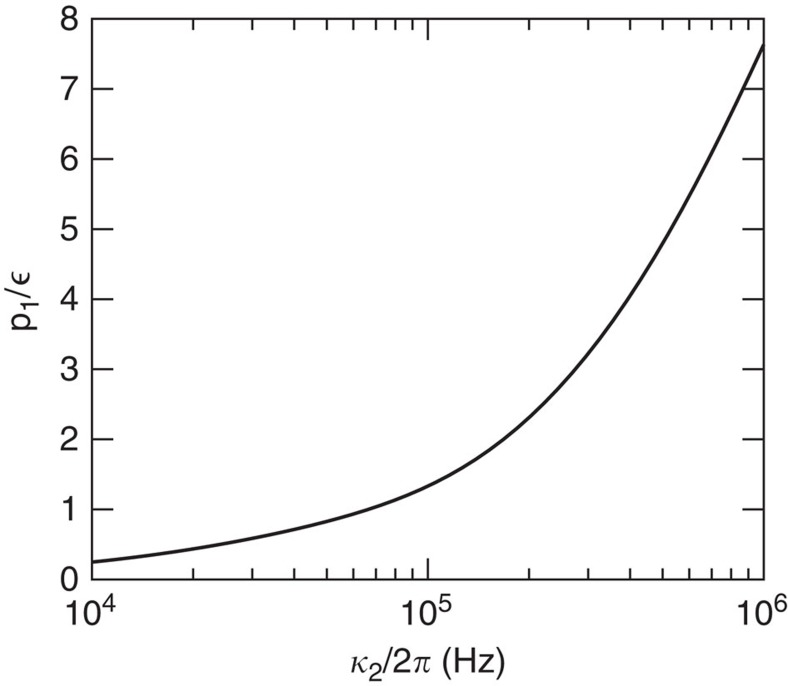
Incoherent photon contribution. The probability to read out incoherent photons (*p*_1_) normalized by the fraction of atoms (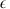) that have been incoherently transferred to the readout state (|1〉) as a function of the linewidth, *κ*_2_ of the filter cavity. 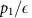 essentially only depends on 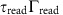 and *κ*_2_ for the parameters that we are considering, which are 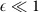, an optical depth of 168 and a finesse of the cell cavity in the range 20–100. Furthermore, we have assumed that 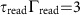, which ensures a temporal filtering of the incoherent photons while keeping a high readout efficiency of the coherent photons. The plot was obtained by numerically simulating the Cs-cells used in the proof-of-principle experiment including the full-level structure of the Cs-atoms.

Atoms can also be in the readout state |1〉 either by inefficient optical pumping or by wall collisions. These atoms will mainly produce ‘incoherent' photons. The incoherent photons will have a much broader temporal and frequency profile than the ‘coherent' photons originating from the symmetric excitation. We can thus to some extent filter them from the coherent photons by sending the light through a filter cavity, which makes a spectral filtering, as well as having a not too long readout time *τ*_read_, which makes a temporal filter. In addition to the incoherent photons, atoms incoherently prepared in the wrong state can also produce coherent photons because the incoherent atoms have an overlap with the symmetric mode. If a fraction 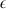 of the atoms are transferred to the state |1〉, the probability to read out a coherent single photon from these atoms is 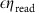. The probability *p*_1_ to read out an incoherent photon can be found to lowest order by assuming that an excitation is stored in any asymmetric mode instead of the symmetric Dicke mode in the perturbative expansion of 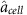 described above. Doing the perturbative expansion, we then get a contribution to *a*_cell_ from these incoherent excitations to the first-order term *a*_1_. From this, we can find the number of incoherent photons in the retrieval. We have evaluated *p*_1_ by numerical simulating the Cs-cells as for the readout (see Supplementary Methods). Figure 5 shows 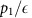 as a function of the linewidth (*κ*_2_) of the filter cavity. We have assumed that 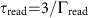 as in Fig. 3b. Note that this choice of readout time ensures a high readout efficiency while still making a temporal filtering of the incoherent photons since these have a smaller readout rate and hence predominantly arrive later. It is seen that 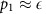 for *κ*_2_≈2*π*·80 kHz. With a linewidth of the filter cavity more narrow than this, the error will thus be dominated by the coherent photons which are emitted with a probability 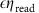. Imposing this linewidth of the filter cavity for the numerical example for the readout efficiency given above for a readout time of *t*=200 μs, would make it drop from ≈90% to ≈86%. Hence, we lose only a little on the readout efficiency by filtering out the incoherent photons. Experimentally, it will be simpler to use the same filter cavity for the retrieval as for the write process, and hence it may be desirable to use a more narrow filter cavity to have an efficient write process (see Fig. 3b). In this case one can use a longer read out time 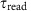 to suppress loss from the filter cavity. After filtering out the incoherent photons, the remaining error is caused by coherent photons from atoms being incoherently prepared in the wrong state. This error is equal to the probability 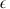 that an atom is in the wrong state.

## Discussion

In conclusion, we have developed a theory for motional averaging for discrete variable systems and proposed an efficient and scalable single-photon source based on atomic ensembles at room temperature. We have considered a specific setup where the atomic ensemble is kept in a small cell inside a cavity and shown how both read and write efficiencies above 90% can be achieved for a real experimental system based on Cs-atoms. The write and read processes have a timescale of 100–200 μs, which is considerably shorter than the demonstrated quantum memory time of 10 ms (ref. 28). To verify the essential effect described by the theory, we have performed a proof-of-principle experiment with room temperature Cs atoms contained in a microcell with spin-preserving coating deposited on the walls. The measurement of the scattered light reveals long coherence time at the single-photon level, resulting in a narrow peak, which is in excellent agreement with the theoretical model being used. This thus confirms the essential feature of the theory.

The room temperature cells considered here provide a promising building block for future quantum networks because of their scalability compared with cold atomic ensembles. As a particular application, we have considered a basic step of DLCZ quantum repeater with a single entanglement swap and a distance of 80 km assuming a dark count rate of 1 Hz and single-photon detection efficiency of 95% (refs 30, 31). Including various experimental imperfections such as intra-cavity losses and inefficient in/out coupling of the cavities, we estimate that a pair with fidelity ∼80% with a Bell state can be distributed at a rate of ∼0.2 Hz (see Supplementary Methods and Supplementary Fig. 5). In this estimate, we have neglected effects from limited memory time and have assumed that a fraction of 0.5% of the atoms have been incoherently transferred to the state |1〉. Note, however, that the rate of entanglement distribution can be greatly enhanced using spatially multiplexing schemes, which are possible because of the scalable nature of the room temperature cells. A particularly attractive feature of such multiplexing is that it also decreases the necessary memory time^22^, and thus relaxes one of the most challenging requirements for long distance communication based on atomic ensembles. The microcells introduced here may thus serve as an essential building block for future photonic networks. On the other hand, for more near term applications the scalable nature of the setup will also be highly interesting for applications requiring multiple single-photon inputs such as for instance photonic quantum simulators^19^,^20^,^21^.

## Methods

### Write process

From the Hamiltonian in equation (1), we obtain the following equations of motion:


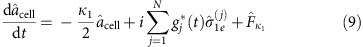



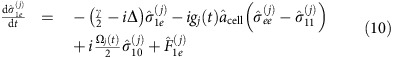



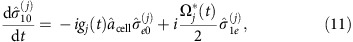


where we have included the cavity intensity decay with a rate *κ*_1_ and the spontaneous emission of the atoms with a rate γ. Associated with these decays, are corresponding Langevin noise operators, 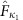, for the cavity decay and 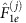 for the atomic decay^29^. Note, that we have neglected dephasing of the atoms, for example, due to collisions. We assume that all the atoms are initially in the ground state |0〉 and that the interaction with the light is a small perturbation to the system. We can therefore assume that 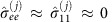. The noise operators describe vacuum noise and will never result in either an atomic or field excitation. Hence, they will never give rise to clicks in the detector (see Fig. 1b) and we can consequently ignore them as described in ref. 29. Furthermore, we treat 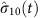 as slowly varying in time and formally integrate equations (9) and (10) to obtain the field operator inside the cell


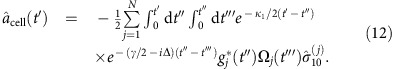


To find the field at the detector, we need to propagate the field through the filter cavity. The input/output relations for the filter cavity are


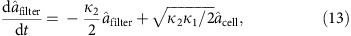



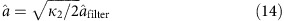


with *κ*_2_ being the intensity decay rate of the filter cavity, 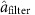 describes the field inside the filter cavity and 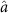 describes the field at the detector. We have again neglected any input noise from the cavity decay since it never gives a click in our detector and we have also neglected intra-cavity losses. Formally integrating equation (13), and using equation (14), gives equations (2) and (3) in the main text.

To evaluate |〈*θ*_*j*_(*t*)〉_e_|^2^ and 〈|*θ*_*j*_(*t*)|^2^〉_e_, we explicitly include the spatial dependence of the couplings assuming that 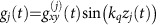 and 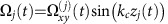, where


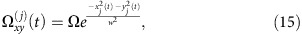



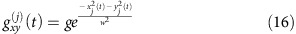


with *w* being the waist of the beams and (*x*_*j*_, *y*_*j*_, *z*_*j*_) is the position of the *j*'th atom. The transverse *xy*-dependence is assumed to be Gaussian while the *z* dependence is sinusoidal due to the standing wave in the cavity. *k*_*q*_ (*k*_*c*_) is the wave vector associated with the quantum photon (classical field). We have neglected additional geometric phases in the gaussian couplings since we are always considering the product 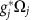.

The cavity field is a standing wave along the *z* direction and both modes are assumed to have a node at the centre of the cell at *z*=0. This geometry ensures an ideal overlap between the two modes at the position of the microcell at the centre of the cavity. Away from the centre of the cavity, the overlap of the two modes is degraded, which can lead to a detrimental phase difference if the length of the microcell along the cavity axis is too long. We estimate that for the Cs-cells used in the proof-of-principle experiment, the write efficiency is only degraded by a factor ∼0.97 for a cell length of ∼1 cm (see Supplementary Methods and Supplementary Fig. 1).

To suppress the effect of Doppler broadening of the atomic levels, Δ will be in the GHz range whereas the transverse waist of the beam will be on the order 50 μm. Consequently, the transverse coupling and the velocity of an atom can be considered constant for the integration over 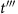 appearing in *θ*_*j*_(*t*), which will have a typical timescale of 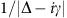. The *z* dependence of the coupling, however, varies rapidly because of the standing wave in the cavity and cannot be assumed to be constant. Writing 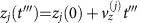, where 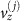 is the *z*-component of the velocity of the *j*'th atom, we can thus perform the integration over 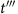 and adiabatically eliminate the optical coherence since we are far detuned. To obtain an expression for |〈*θ*_*j*_(*t*)〉_e_|^2^, we assume that the spatial distribution of the atoms is uniform and that the velocity distribution of the atoms follows the Maxwell–Boltzmann distribution with temperature *T*. Both distributions are assumed to be independent of time. In our analytical calculations, we also assume 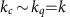 and that 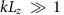 such that 〈*e*^±2*ikz*^〉≈0, but in our numerical simulations, we set the difference between *k*_*c*_ and *k*_*q*_ corresponding to the real level structure of Cs where a splitting between |0〉 and |1〉 is 9.2 GHz. Here, 2*L*_*z*_ is the length of the cell in the beam direction. With these assumptions, we obtain


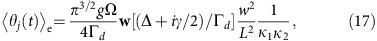


where we have assumed that 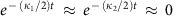. Furthermore, we have assumed that the cell dimensions (*x* × *y* × *z*) are 2*L* × 2*L* × 2*L*_*z*_ and that 
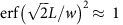
 meaning that we ignore any small portion of the beam, which is outside the cell. **w**[…] is the Faddeeva function defined as 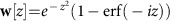 and 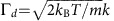 is the Doppler width of the atomic levels at the temperature *T* where *m* is the atomic mass and *k*_B_ is the Boltzmann constant.

We evaluate 〈|*θ*_*j*_(*t*)|^2^〉_e_ under similar assumptions for the atoms as presented above. In the simplified model used in the main text, we assumed that the decay of the correlations is exponential such that, for example, 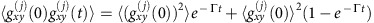. We substantiate this assumption by simulating a box of randomly moving, non-interacting atoms and find good agreement with a decay rate Γ=*αv*_thermal_/*w* where *v*_thermal_ is the average thermal velocity of the atoms, *w* is the waist of the Gaussian cavity mode and *α* is a numerical constant on the order of unity (see Supplementary Methods and Supplementary Fig. 3). Employing this model for the atomic correlations and assuming 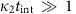 such that the effective interaction time (1/*κ*_2_) is set by the linewidth of the filter cavity, we find


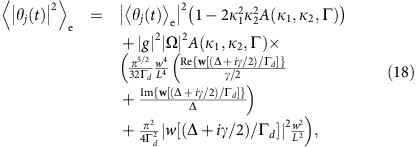


where we have defined


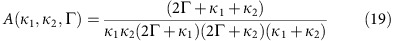


and we have neglected all terms 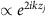, since these average to zero rapidly. Using equations (17) and (18), we can directly evaluate *η*_write_ from equation (4). In the limit of 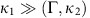 and 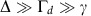, the expression for *η*_write_ reduces to equation (5) in the main text.

### Proof-of-principle

Here we describe some of the experimental details of the proof-of-principle experiment. We refer to Fig. 4 for a sketch of the experimental setup. The light is red-detuned by 2.8 GHz from the 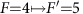 D2 transition. For this detuning, the polarization state of the probe light is affected by Cs atoms in both *F*=4 and *F*=3 ground-state manifolds. The atomic ensemble is contained in a glass-cell with dimensions 300 μm × 300 μm × 1 cm corresponding to an average wall-to-wall time of flight of ∼1.4 μs. The walls of the cell are covered with an alkene coating^26^,^27^, resulting in longitudinal and transverse spin lifetime in the dark *T*_1_≈17 ms and *T*_2_≈10 ms, respectively. The atomic density inside the cell is estimated to be ∼8 × 10^−10^ cm^−3^ (ref. 28). The cell is placed inside a standing wave optical cavity to enhance the light–matter interaction. The cavity has finesse 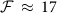, determined by the output coupler (intensity reflection *R*_2_≈80%) and the optical losses in the cell; currently the light intensity loss in the cell is ∼13% per roundtrip, limited by the deterioration of the anti-reflection coating of the walls during the cell fabrication. A Pound–Drever–Hall technique is used to lock the cavity on resonance. The cavity mode has ≈55 μm waist radius, which is a compromise between the requirement for strong coupling of light to the atomic ensemble and the requirement for low propagation losses through the cell. A small portion of the beam at the cavity output is used in a feedback loop to compensate for the probe-intensity drift and maintain the same photon shot noise during the time of measurement.

The measurement is performed on atoms in approximately their thermal state, that is, the atoms are randomly distributed in the 16 magnetic sublevels of the *F*=3 and *F*=4 hyperfine manifold. There is a small deviation from the thermal state due to weak optical pumping from the probe, and all measurements are recorded in the resulting steady state. In this case, there is no macroscopic orientation and the probe induced back-action noise is negligible. The polarimetry noise is the sum of the photon shot noise and the Raman scattered photon noise. The photon shot noise has a white power spectrum, whereas the spectrum of the recorded Raman noise is centred around the Larmor frequency due to the energy difference between magnetic sublevels. We perform Raman noise measurements for two different Larmor frequencies, ∼0.8 and ∼2.6 MHz, at the same probe power. By subtracting the two power spectra, the photon shot noise and the electronic noise contribution to the recorded spectra can be removed and the Raman noise is acquired.

### Readout

From Heisenberg's equations of motion, we obtain


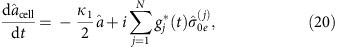



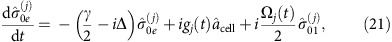



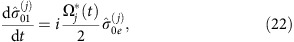


where we have assumed that 
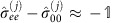
 and that the dynamics of 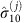 are governed by the classical drive (Ω). Furthermore, we have neglected the noise operators associated with spontaneous emission and cavity decay, as in the write process. We can formally integrate equation (21), assuming the *xy*-dependence of the couplings to be constant for the integration while the *z*-dependent parts are of the form 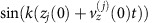, as in the write process. The integration gives a set of coupled equations


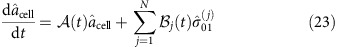



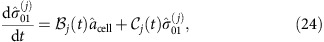


where


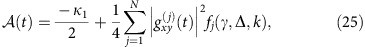



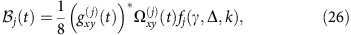



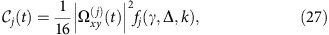



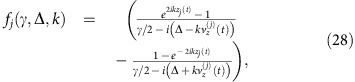


and we have assumed that *k*_*c*_≈*k*_*q*_≈*k*. The equations can be expressed as a matrix system of the form


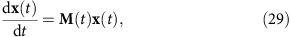


where 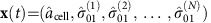 and **M**(*t*) is the coupling matrix between the atoms and the cavity field. We now split the couplings into (large) average time-independent parts, 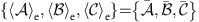, and (small) time-dependent parts, 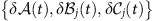. The coupling matrix can then be expressed as **M**(*t*)=**M**_0_+*δ***M**(*t*) where **M**_0_ contains the average time-independent couplings, while *δ***M**(*t*) contains the time-dependent fluctuations. As described in the main text, we can get a perturbative expansion of 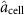 from equation (29). To lowest order, we find that


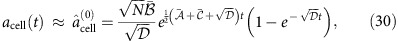


where 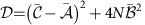. Inserting equation (30) in equation (7) and taking the limit of Ω→0 and 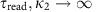, gives the zeroth-order readout efficiency in equation (8). Note that from equation (30), we identify the readout rate 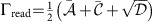.

## Additional information

**How to cite this article:** Borregaard, J. *et al*. Scalable photonic network architecture based on motional averaging in room temperature gas. *Nat. Commun.* 7:11356 doi: 10.1038/ncomms11356 (2016).

## Supplementary Material

Supplementary Information Supplementary Figures 1-5, Supplementary Methods and Supplementary References.
