# Supplementary material for: Scalable photonic network architecture based on motional averaging in room temperature gas
Source: Nat Commun. 2016 Apr 14;7:11356. doi: 10.1038/ncomms11356 (PMC4834638; doi:10.1038/ncomms11356)
Supplement: Supplementary Information — Supplementary Figures 1-5, Supplementary Methods and Supplementary References. [file ncomms11356-s1.pdf]

Supplementary Figures

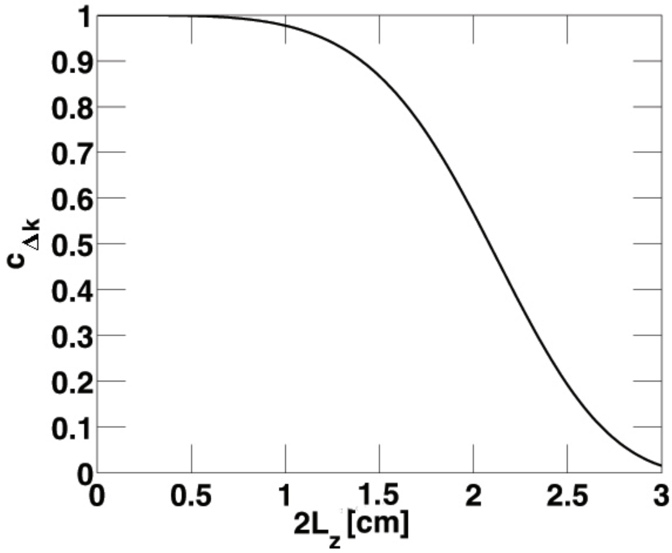

Supplementary Figure 1. Limitation in efficiency, $c_{\Delta k}$, from the difference in wavenumbers as a function of the cell length $2L_z$ for $\Delta_k \approx 193$ m^{-1} corresponding to the $2\pi \cdot 9.2$ GHz splitting between the hyperfine ground states of ^{133}Cs. The atoms are assumed to be evenly distributed in the cell. $c_{\Delta k} \gtrsim 0.97$ for $2L_z \lesssim 1$ cm.

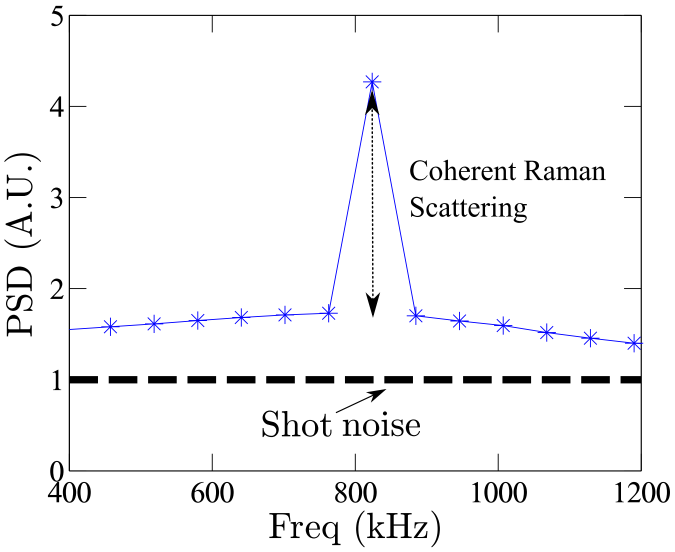

Supplementary Figure 2. Experimental data of the power spectral density (PSD) of the Raman scattered light measured in the proof-of-principle experiment. These data were obtained for a pulse length of ~ 16 μs. The electronic noise has been removed from the data and the shot noise has been normalized to unity. The number of coherent Raman-scattered photons, equally distributed in the Stokes and anti-Stokes lines, can be estimated by the comparing the height of the central peak (dotted line) to the shot noise of light. For the proof of principle experiment, the number of coherently scattered photons in the antiStokes sideband over the 16 μs of the pulse is estimated to be ~ 1.2 photons.

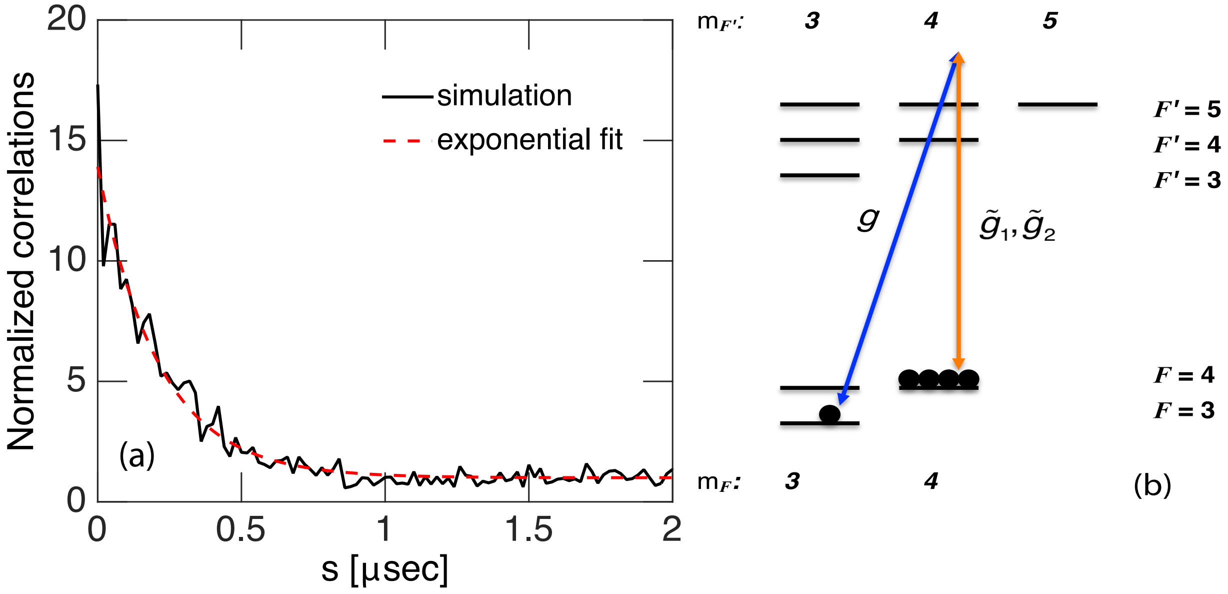

Supplementary Figure 3. (a) Simulation of the correlations $\langle XY, Z\rangle_e(s)$. The correlations are normalized to be unity for $s \to \infty$ where there are no correlations and $\langle XY, Z\rangle_e(s) \to |\langle XY\rangle_e|^2\,|\langle Z\rangle_e|^2$. The data from the simulation have been fitted with an exponential model validating our assumption of an exponential decay of the correlations. The fit gives a decay rate of $\Gamma = 2\pi{\cdot}0.75$ MHz corresponding to $\Gamma \sim 1.3v_{\text{thermal}}/w$. (b) Sketch of the $6^2S_{1/2}$ and $6^2P_{3/2}$ hyperfine levels in ^{133}Cs. A Λ-atom is realized with $|0\rangle = |F = 4, m_F = 4\rangle$, $|1\rangle = |F = 3, m_F = 3\rangle$ as ground states in $6^2S_{1/2}$ and $|e_1\rangle = |F' = 4, m_{F'=4}\rangle$ as the excited level in $6^2P_{3/2}$. To characterize the optical depth, we consider the transitions $|0\rangle \to |e_1\rangle$ and $|0\rangle \to |e_2\rangle = |F' = 5, m_{F'} = 4\rangle$ characterized by $\tilde{g}_1$ and $\tilde{g}_2$, respectively.

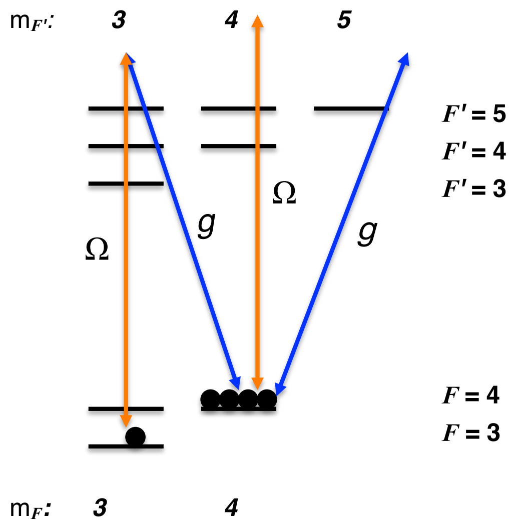

Supplementary Figure 4. Schematic view of the readout realized in the hyperfine levels of $6^2S_{1/2}$ and $6^2P_{3/2}$ in ^{133}Cs. We imagine the single exitation to be stored in state $|F = 3, m_F = 3\rangle$ while the macroscopically populated state is $|F = 4, m_F = 4\rangle$. Note that the classical drive also couples $|F = 4, m_F = 4\rangle$ to $|F' = 4, m_{F'} = 4\rangle$ and $|F' = 5, m_{F'} = 4\rangle$, which can pump atoms out of $|F = 4, m_F = 4\rangle$. These couplings are however sufficiently suppressed by the large splitting of $2\pi \cdot 9.2$ GHz between the ground states.

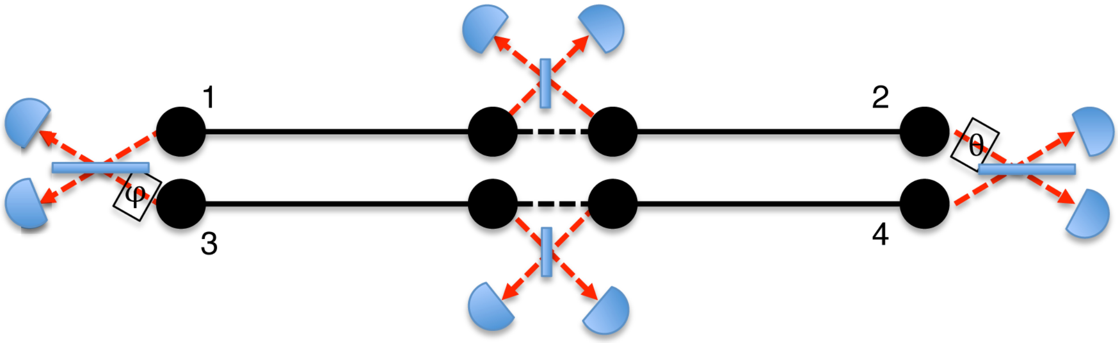

Supplementary Figure 5. Sketch of the postselection procedure after a single step of entanglement swapping (middle stations). The entanglement swaps creates entanglement between ensembles 1,2 and 3,4, respectively. Ensembles 1,2,3 and 4 are then read out and we condition on a single click at both stations. The phases ϕ and θ are ideally equal.

.

Supplementary Methods

Numerical simulation - Write. To justify our assumption of an exponential decay of the correlations appearing in $\langle|\theta_j(t)|^2\rangle_{\rm e}$ and to qualitatively characterize the write efficiency, we perform a numerical simulation of a gas of non-interacting atoms in a cell. We have based the simulation on the microcells filled with Cs-atoms, which were used in the proof-of-principle experiment. These cells have dimensions of 300 μm $\times$ 300 μm $\times$ 1 cm. The cells have been placed inside a cavity with a linewidth of $\kappa_1 \approx 2\pi\cdot 46$ MHz and both the field from the quantum photon and the classical drive are assumed to have approximately a Gaussian shape with a waist of 55 μm. The small beam waist ensures that we can neglect cavity losses from the walls of the cell. An approximate Λ-atom can be realized in the hyperfine states of Cs with state $|0\rangle = |F=4, m_F=4\rangle$ and state $|1\rangle = |F=3, m_F=3\rangle$ in the $6^2S_{1/2}$ ground state manifold. The Doppler width of the atomic levels is $\Gamma_d \sim 2\pi\cdot 225$ MHz at a temperature of $T = 293$ K and we assume a detuning of $\Delta \sim 4\Gamma_d$ from the excited level such that the effect of Doppler broadening is negligible.

Starting from Eq. (3) in the article and performing the integral over t''' as described in the Methods section in the article, we can express $\langle|\theta_j(t)|^2\rangle_{\rm e}$ as

$$\langle|\theta_j(t)|^2\rangle_{\rm e} = \frac{1}{16}\int_0^t dt_1' \int_0^{t_1'} dt_1'' \int_0^t dt_2' \int_0^{t_2'} dt_2'' e^{-\kappa_2/2(t-t_1')} e^{-\kappa_1/2(t_1'-t_1'')} e^{-\kappa_2/2(t-t_2')} e^{-\kappa_1/2(t_2'-t_2'')} \times\langle XY_j^*(t_1'')XY_j(t_2'')Z_j^*(t_1'')Z_j(t_2'')\rangle_{\rm e}, \quad (1)$$

where we have defined

$$Z_j(t) = \frac{e^{i(\Delta_k)z_j(t)} - e^{-i(k_c+k_q)z_j(t)}}{-\gamma/2 + i(\Delta + k_c v_z^{(j)}(t))} + \frac{e^{-i(\Delta_k)z_j(t)} - e^{i(k_c+k_q)z_j(t)}}{-\gamma/2 + i(\Delta - k_c v_z^{(j)}(t))}, \quad (2)$$

$$XY_j(t) = \Omega g e^{\frac{-2x_j^2(t) - 2y_j^2(t)}{w^2}}. \quad (3)$$

Note that we have not made the assumption of $k_c \approx k_q \approx k$, as in our analytical calculations, since we have the $2\pi\cdot 9.2$ GHz splitting between the ground states, which corresponds to $k_q - k_c = \Delta_k \approx 193\ {\rm m}^{-1}$. With a cell length of $2L_z = 1$ cm the assumption $2\Delta_k L_z \ll 1$ is close to being violated and we shall therefore not make this assumption. The extra terms $\propto e^{\pm i\Delta_k z_j}$ will approximately result in a factor of

$$c_{\Delta k} = \frac{\langle\cos(\Delta_k z_j)\rangle^2}{\langle\cos(\Delta_k z_j)^2\rangle}, \quad (4)$$

which should be multiplied with the analytical expression for the write efficiency, which was obtained assuming $\Delta_k = 0$. Supplementary Figure 1 shows how $c_{\Delta k}$ depends on the length of the cell assuming that the atoms are equally distributed in the entire cell. It is seen from Supplementary Figure 1 that as long as the length of the cell is $2L_z \lesssim 1$ cm then $c_{\Delta k} \gtrsim 0.97$ for $\Delta_k \approx 193\ {\rm m}^{-1}$ and hence the frequency difference between the quantum and classical fields does not significantly degrade the write efficiency. In all our numerical simulations, we, however, keep the terms $\propto e^{\pm i\Delta_k z_j}$ for completeness.

The correlations appearing in $\langle XY_j^*(t_1'')XY_j(t_2'')Z_j^*(t_1'')Z_j(t_2'')\rangle_{\rm e}$ (see Eq. (1)) depend on the time difference $|t_1''-t_2''|$ and we therefore introduce the shorthand notation $\langle XY_j^*(t_1'')XY_j(t_2'')Z_j^*(t_1'')Z_j(t_2'')\rangle_{\rm e}=\langle XY,Z\rangle_{\rm e}(t_1''-t_2'')$. We change to the variables $u=t_1''+t_2''$ and $s=t_1''-t_2''$ and by changing the order of integration, we can perform the integrals over t,t_1',t_2' and u. To obtain the write efficiency $\eta_{\rm write}$, we need to perform an additional integration over t (see Eq. (4) in the article). We are therefore left with

$$\int_0^{t_{\rm int}}\langle|\theta(t)|^2\rangle_{\rm e}dt=\int_0^{t_{\rm int}}h(t_{\rm int},\kappa_1,\kappa_2,s)\langle XY,Z\rangle_{\rm e}(s)ds, \tag{5}$$

where $h(t_{\rm int},\kappa_1,\kappa_2,s)$ is a function of s obtained by performing the integrals over t,t_1',t_2' and u. We can evaluate the integral over s numerically by simulating the correlations $\langle XY,Z\rangle_{\rm e}(s)$. Since the atoms do not interact with each other, we independently simulate the motion of $N=5000$ atoms through the cell and evaluate the correlations of atoms at points separated in time by s. Finally, we average over many realizations. At room temperature, we assume that the motion of the atoms is classical. Furthermore, we assume that the atoms are evenly distributed in the cell and that their velocity distribution is described by the Maxwell Boltzmann distribution at a temperature $T=293$ K. We assume that the atoms are re-thermalized completely after every collision with the walls of the cell but qualitatively similar results are obtained for a ballistic model without thermalization. For the ballistic model, the Z_j parts of the couplings in principle do not average down. This could lead to effects not averaged away by using narrow filter cavities. However, we are far detuned compared to the Doppler width of the atoms and the cavity fields are standing waves, which can be viewed as the superposition of two counter propagating waves. As a result, the effect of the velocity fluctuations of the atoms cancel and the fluctuations in the Z_j terms are greatly suppressed. This is in contrast to what happens in ensemble based schemes with a laser coming from one side where Doppler effects do not go away by working far off resonance [1]. Consequently, we expect similar results for the ballistic model as for the model with complete thermalization. We have explicitly verified this by repeating our simulations with the ballistic model, which lead to more or less identical results as for the model with thermalization (the two simulations cannot be distinguished due to the noise from the random nature of the simulations). The result of a simulation with thermalization is seen in Supplementary Figure 3a, which shows how the correlations decay as a function of s such that for $s\to\infty$, we have $\langle XY,Z\rangle_{\rm e}(s)\to|\langle XY\rangle_{\rm e}|^2|\langle Z\rangle_{\rm e}|^2$. This enables us to introduce a maximal cutoff, $s_{\rm max}$, in the numerical integral appearing in Eq. (5), above which, the correlations have effectively vanished. As a result, we can semianalytically evaluate $\eta_{\rm write}$ for an arbitrary pulse length $t_{\rm int}$ without additional numerical difficulty. Note that Supplementary Figure 3a also shows that the exponential model of the decay of the correlations assumed in our analytical calculations is a good approximation.

Based on our simulations of $\langle XY,Z\rangle_{\rm e}(s)$, we have also estimated the power spectral density, PSD in the proof of principle experiment. The PSD was measured (see Fig. 2 in the article and Supplementary Figure 2) by measuring the Faraday rotation of light transmitted through the cell. In these measurements, the light is emitted by the scattering between different m_F states in an applied magnetic field. As a result, the signal is modulated at the Larmor precession frequency. We therefore consider the modulated power spectral density

$$\mathrm{PSD}(f)\propto\frac{1}{t_{\rm int}^2}\int_0^{t_{\rm int}}dt\int_0^{t_{\rm int}}dt'\langle XY,Z\rangle_{\rm e}(t-t')e^{2i\pi f(t-t')} \tag{6}$$

$$\propto\frac{1}{t_{\rm int}^2}\int_0^{t_{\rm int}}dt\int_0^{t_{\rm int}}dt'\,|\langle XY\rangle_{\rm e}|^2|\langle Z\rangle_{\rm e}|^2e^{2i\pi f(t-t')}$$
$$+\frac{1}{t_{\rm int}^2}\int_0^{t_{\rm int}}dt\int_0^{t_{\rm int}}dt'(\langle XY,Z\rangle_{\rm e}(t-t')-|\langle XY\rangle_{\rm e}|^2|\langle Z\rangle_{\rm e}|^2)e^{2i\pi f(t-t')} \tag{7}$$

$$\propto\delta_{f,0}+\frac{1}{t_{\rm int}^2}\int_0^{t_{\rm int}}dt\int_0^{t_{\rm int}}dt'(\langle XY,Z\rangle_{\rm e}(t-t')-|\langle XY\rangle_{\rm e}|^2|\langle Z\rangle_{\rm e}|^2)e^{2i\pi f(t-t')} \tag{8}$$

where f is the frequency and $\delta_{f,0}$ is the Kronecker delta function. Note that we have assumed that the coherent light-atom interaction gives a contribution of $\frac{1}{t_{\rm int}^2}\int_0^{t_{\rm int}}dt\int_0^{t_{\rm int}}dt'\,|\langle XY\rangle_{\rm e}|^2|\langle Z\rangle_{\rm e}|^2e^{2i\pi f(t-t')}=\delta(f)$ to the PSD. In the proof-of-principle experiment, the atoms are subject to a magnetic field, which makes the atomic spins precess around the mean spin direction with a Larmor frequency of 823.8 kHz. The Raman scattered part of the PSD is thus centered around this frequency. However, the measured PSD also contains both the shot noise of the light and electronic noise from the measurement equipment. Since we are only interested in the signal from the atomic interaction, this noise is isolated by performing a second measurement at a higher Larmor frequency (2594 kHz) and subtract it from the first measurement. The higher Larmor frequency is chosen such that the two atomic signals are well separated in frequency. In the simulated PSD, we have fitted a Lorentzian to the broad feature centered at 823.8 kHz and shifted

it to be centered at 2594 kHz. This has then been subtracted from the simulated data to include the substraction of the two signals in the experiment.

To validate that the proof-of-principle experiment is probing the theory in the right limit of single photon Raman scattering, we estimate the number of Raman photons scattered over the relevant pulse length. This number can be found from the ratio of the height of the central peak in the PSD to the shot noise of light. In the experiment, the balanced polarimetry measures the amplitude quadrature of the field in the polarisation axis perpendicular to the drive field $\hat{a}_{\rm sc}$. Neglecting proportionality factors irrelevant for this calculation, the PSD at a discrete frequency f is given by: $\mathrm{PSD}(f) \propto \tilde{\mathcal{X}}^\dagger(f)\tilde{\mathcal{X}}(f)$, where:

$$\tilde{\mathcal{X}}(f) \propto \int_0^{t_{\rm ms}} \left[\hat{a}_{\rm sc}(t) + \hat{a}_{\rm sc}^\dagger(t)\right] e^{2i\pi f t} dt = \hat{a}_{\rm sc}(f) + \left[\hat{a}_{\rm sc}(-f)\right]^\dagger . \tag{9}$$

Here $\hat{a}_{\rm sc}(f) = \int_0^{t_{\rm ms}} \hat{a}_{\rm sc}(t) e^{2i\pi f} dt$ and $t_{\rm ms}$ is the measurement time. It can then be shown that the PSD takes the form:

$$\mathrm{PSD}(f) \propto 1 + \left[\hat{a}_{\rm sc}(-f)\right]^\dagger \left[\hat{a}_{\rm sc}(-f)\right] + \left[\hat{a}_{\rm sc}(f)\right]^\dagger \left[\hat{a}_{\rm sc}(f)\right] . \tag{10}$$

The unity term in the left-hand part of the above relationship is the photon shot noise contribution, present even when there is no scattered field; the second and third terms represent the Raman-scattered number of photons in the lower (Stokes line) and upper (anti-Stokes line) sideband respectively. In Supplementary Figure 2, the PSD recorded with a pulse duration of $t_{\rm ms} \approx 16$ μs is plotted. The peak height at the Larmor frequency due to the Raman scattering has equal contributions from the Stokes and the anti-Stokes lines. The number of coherently scattered photons during the measurement pulse is the ratio of the peak height excluding the photon shot noise and the incoherent photons (denoted with a dotted lines in Supplementary Figure 2) to the photon shot noise level, which corresponds to a power of one photon per unit bandwidth as shown in Eq. (10). From Supplementary Figure 2, we find that for the proof-of-principle experiment approximately 1.2 photons are coherently scattered in the Stokes line during the 16 μs pulse. This corresponds to approximately 8 photons over a pulse duration of 106 μs that can lead to an efficient write step according to our proposal. Although this is slightly higher than the single photon required for the protocol, we expect the spectrum to be independent of intensity at these light levels.

In the above calculation, it is important to estimate the photon shot noise level. For this, the electronic noise, as measured with no light at the detector, is removed from the recorded PSD and the spectrum is corrected for the detector frequency response. The photon shot noise then corresponds to the level where the PSD levels off at frequencies a few linewidths of the broad Lorentzian away from the Larmor frequency; at these frequencies the power from the Raman scattered photons is negligible and the PSD is determined only by the photon shot noise. The photon shot noise is also verified by performing balanced polarimeter detection of light that has not interacted with the atomic ensemble and with power equal to the one as the drive light in the experiment. We note that the detector response was characterized by such a measurement.

Number of photons. As mentioned in the main text, the purpose of the filter cavity is both to increase the averaging time and to filter the quantum photon from the classical photons. We will now estimate the number of classical photons, which needs to be filtered from the single quantum photon. In order to do this, we need to characterize the ensemble, which we do by introducing the optical depth d.

To obtain an expression for the optical depth, we assume that we are working with the previously mentioned Cs-cells. The relevant level structure is shown in Supplementary Figure 3b.

In the write process, the classical drive is applied on the transitions between the ground state $|0\rangle = |F = 4, m_F = 4\rangle$ and the excited states $|e_1\rangle = |F' = 4, m_{F'} = 4\rangle$ and $|e_2\rangle = |F' = 5, m_{F'} = 4\rangle$ characterized by $\tilde{g}_1$ and $\tilde{g}_2$, respectively. The quantum photon is created on the transition $|e_1\rangle \to |1\rangle = |F = 3, m_F = 3\rangle$ characterized by g. Note that with this field configuration, the cell-cavity in principle also mediate the transition $|F = 4, m_F = 4\rangle \to |F = 4, m_F = 3\rangle$ in the write setup but this transition is suppressed by the $2\pi \cdot 9.2$ GHz splitting between the ground states, which makes the corresponding photon non-resonant with the subsequent filter-cavity. This transition will, therefore, never give a click in the detector. Since the interaction is only a perturbation to the system, we can therefore neglect this transition in our numerical simulations. All atoms in the ensemble are initially pumped to the ground state $|0\rangle$ and, in order to characterize the optical depth, we assume that the transition characterized by g is non-driven and ignore any cavity (Purcell) enhancement of the corresponding decay. The cavity field thus only couples $|0\rangle \to |e_1\rangle$ and $|0\rangle \to |e_2\rangle$ with coupling constants $\tilde{g}_1$ and $\tilde{g}_2$, respectively (see Supplementary Figure 3b).

The equations of motion for the cavity field, $\hat{a}_{\rm cav}$, and the relevant atomic operators in a suitable rotating frame

are

$$\dot{\hat{a}}_{\rm cav} = -(\kappa/2)\hat{a}_{\rm cav} + i\sum_{j=1}^{N}\left[\tilde{g}_1^{(j)}(t)\hat{\sigma}_{e_10}^{(j)} + \tilde{g}_2^{(j)}(t)\hat{\sigma}_{e_20}^{j}\right] \tag{11}$$

$$\dot{\hat{\sigma}}_{e_10}^{(j)} = -(\gamma_1/2 - i\Delta_1)\hat{\sigma}_{e_10}^{(j)} + i\tilde{g}_1^{(j)}(t)\hat{a}_{\rm cav} \tag{12}$$

$$\dot{\hat{\sigma}}_{e_20}^{(j)} = -(\gamma_2/2 - i\Delta_2)\hat{\sigma}_{e_20}^{(j)} + i\tilde{g}_2^{(j)}(t)\hat{a}_{\rm cav}, \tag{13}$$

where $\sigma_{e_l0}^{(j)} = |e_l\rangle_j\langle 0|$ $(l = 1, 2)$ and we have assumed that $\sigma_{e_le_l}^{(j)} - \sigma_{00}^{(j)} \approx -1$. For simplicity, we have assumed the couplings ($\tilde{g}$) to be real. $\Delta_1 = \omega_1 - \omega_{\rm cav}$ ($\Delta_2 = \omega_2 - \omega_{\rm cav}$) is the detuning of $|e_1\rangle$ ($|e_2\rangle$), while γ_1 (γ_2) is the corresponding decay rate. Here ω_1 (ω_2) is the frequency associated with the atomic level and $\omega_{\rm cav}$ is the frequency of the cavity field. Formally integrating Eqs. (12)-(13), assuming that $\sigma_{e_10}^{(j)} = \sigma_{e_20}^{(j)} = 0$ at time $t = 0$, and inserting the resulting expressions for $\sigma_{e_10}^{(j)}$ and $\sigma_{e_20}^{(j)}$ into Eq. (11) gives

$$\dot{\hat{a}}_{\rm cav} = -(\kappa/2)\hat{a}_{\rm cav} - \sum_{j=1}^{N}\left[\tilde{g}_1^{(j)}(t)\int_0^t e^{-(\gamma_1 - i\Delta_1)(t-t')}\tilde{g}_1^{(j)}(t)\hat{a}_{\rm cav}(t'){\rm d}t' + \tilde{g}_2^{(j)}(t')\int_0^t e^{-(\gamma_2 - i\Delta_2)(t-t')}\tilde{g}_2^{(j)}(t')\hat{a}_{\rm cav}(t'){\rm d}t'\right], \tag{14}$$

where we have explicitly written the time dependence of $\hat{a}_{\rm cav}$ inside the integrals. We evaluate the integrals in Eq. (14) assuming that we can treat $\hat{a}_{\rm cav}(t')$ as a constant in time and move it outside the integrals. Furthermore, we assume that $\tilde{g}_l^{(j)}(t') = \tilde{g}_{l,xy}^{(j)}(t)\sin(k(z_j(0) + v_z^{(j)}(0)t'))$ similar to the procedure described in the article. Note that k is the wavenumber associated with the cavity field while $z_j(0)$ ($v_z^{(j)}$) is the z-part of the position (velocity) of the j'th atom. After evaluating the integrals, we obtain

$$\dot{\hat{a}}_{\rm cav} = -(\kappa/2)\hat{a}_{\rm cav} + \frac{\hat{a}_{\rm cav}}{4}\sum_{j=1}^{N}\left[\left|\tilde{g}_{1,xy}^{(j)}(t)\right|^2 Z_j(\Delta_1,\gamma_1,k) + \left|\tilde{g}_{2,xy}^{(j)}(t)\right|^2 Z_j(\Delta_2,\gamma_2,k)\right], \tag{15}$$

where we have adiabatically eliminated the optical coherence and have rewritten $Z_j(t)$ defined in Eq. (2) to

$$Z_j(\Delta,\gamma,k) = \frac{e^{2ikz_j(t)} - 1}{\gamma/2 + i(kv_j(0) - \Delta)} - \frac{1 - e^{-2ikz_j(t)}}{\gamma/2 - i(kv_j(0) + \Delta)}, \tag{16}$$

such that γ, Δ, and k become variable parameters. We now perform an ensemble average of Eq. (15) assuming that the atoms are evenly distributed in the cell and that their velocity distribution follows a Maxwell Boltzmann distribution, as previously considered. Furthermore, we assume that the xy-dependence of the couplings are Gaussians similar to Eqs. (15)-(16) in the Methods section in the article and that we are detuned far from the Doppler width of the atoms. This results in

$$\dot{\hat{a}}_{\rm cav} = -(\kappa/2)\hat{a}_{\rm cell} - \frac{\hat{a}_{\rm cell}N}{4}\left[\frac{|\tilde{g}_1|^2\gamma_1}{\gamma_1^2/4 + \Delta_1^2} + \frac{|\tilde{g}_2|^2\gamma_2}{\gamma_2^2/4 + \Delta_2^2}\right]\frac{\pi}{8}\frac{w^2}{L^2} + i[\ldots], \tag{17}$$

where the imaginary part is contained in $[\ldots]$. The second term in Eq. (17) is identified as the single pass optical depth, $\tilde{d}$, divided by the cavity round trip time, τ, where $\exp(-\tilde{d})$ is the attenuation of the light field after passing through the ensemble. Since $\tilde{d}$ depends on, e.g., the detuning, it is not a direct characterisation of the ensemble. Instead, in analogy with Ref. [2], we characterise the ensemble by d, the hypothetical optical depth, which would be obtained for resonant fields in the absence of Doppler broadening and hyperfine interaction, i.e., Eq. (17) with $\Delta_1 = \Delta_2 = 0$. Furthermore, we assume that $\gamma_1 = \gamma_2 = \gamma$ such that the optical depth is

$$d = \frac{N\tau}{\gamma}\left(|\tilde{g}_1|^2 + |\tilde{g}_2|^2\right)\alpha_{xy} \tag{18}$$

where we have defined the factor $\alpha_{xy} = \frac{\pi}{8}\frac{w^2}{L^2}$. Note that Eq. (18) can be rewritten to the following well known formula for the optical depth [3]

$$d = 6\pi\frac{N}{(2L)^2}\tilde{\lambda}^2\left(\frac{\gamma_{1,0} + \gamma_{2,0}}{\gamma}\right), \tag{19}$$

where $2L$ is the transverse size of the cell, $\tilde{\lambda} = \lambda/2\pi$ is the rescaled wavelength of the light, and $\gamma_{s,0}$ is the spontaneous decay rate of level e_s back to $|0\rangle$ (s=1,2). The optical depth can also be related to the Faraday rotation angle, θ_F, which is typically measured in experiments and used to estimate the number of atoms, N in the ensemble [4, 5]. For ^{133}Cs, the relation between θ_F and N is [5]

$$N = \left| \frac{32\pi L^2 \theta_F \Delta_2}{a_1(\Delta_2)\gamma\lambda^2} \right| \tag{20}$$

where $a_1(\Delta_2)$ is the vector polarisability given by

$$a_1(\Delta_2) = \frac{1}{120}\left(-\frac{35}{1-\Delta_{3'5'}/\Delta_2} - \frac{21}{1-\Delta_{4'5'}/\Delta_2} + 176 \right), \tag{21}$$

with $\Delta_{x'5'}$ denoting the hyperfine splitting between level $F' = x$ and $F' = 5$. Combining Eq. (19) and Eq. (20) gives the following relation between d and θ_F

$$d = \left| \frac{12\Delta_2\theta_F}{a_1(\Delta_2)} \frac{\gamma_{1,0}+\gamma_{2,0}}{\gamma^2} \right| \tag{22}$$

For the cells used in the proof-of-principle experiment, the Faraday rotation angle has been measured to be 4.4^{o} for a detuning of $\Delta_2 = 2\pi \cdot 850$ MHz. This translates into an optical depth of $d \approx 168$.

Having defined the optical depth, we can now estimate the number of classical photons that need to be filtered from the quantum photon. The field at the detector (see Fig. 1b in the article) is described by the operator $\hat{a}$ in Eq. (2) in the article. Assuming a length, t_{int}, of the write pulse, we estimate the average number of quantum photons, N_{quant} at the detector as

$$N_{\text{quant}} = \langle \hat{a}^\dagger \hat{a} \cdot t_{\text{int}} \rangle = \frac{1}{16}\kappa_2^2\kappa_1 N \langle |\theta_j|^2 \rangle_{\text{e}}, \tag{23}$$

where we have used that $\langle |\theta_j|^2 \rangle_e$ is independent of time as shown in Eq. (18) in the Methods section in the article. Note that $\langle |\theta_j|^2 \rangle_e \propto |g|^2 |\Omega|^2$ and we estimate the number of classical photons contained in the write pulse as $N_{\text{clas}} \sim |\Omega|^2 t_{\text{int}}\kappa_1/(4|\tilde{g}|^2) = |\Omega|^2 t_{\text{int}}\kappa_1/(4\beta|g|^2)$, where $\beta = |\mu_{\tilde{g}}|^2/|\mu_g|^2$ is the ratio between the Clebsch-Gordan coefficients (μ) of the transitions characterized by $\tilde{g}$ and g (see Supplementary Figure 3b). From Eq. (23), we then get

$$N_{\text{clas}} \sim \frac{N_{\text{quant}}}{N} \frac{4}{|g|^4 \langle |\theta_j|^2 \rangle_{\text{e}} \beta\kappa_2^2}. \tag{24}$$

The number of classical photons that needs to be filtered is finally estimated by setting $N_{\text{quant}} = 1$. Using Eqs. (18)-(19), we can express N_{clas} in terms of the optical depth and the finesse of the cell-cavity, defined as $\mathcal{F} = 2\pi/(\tau\kappa_1)$, where τ is the cavity roundtrip time. Furthermore, we assume that $\langle |\theta_j|^2 \rangle_{\text{e}} \approx |\langle \theta_j \rangle_{\text{e}}|^2$ such that the number of classical photons can be estimated as

$$N_{\text{clas}} \sim \frac{8\pi\beta_2^2 L^2 \Delta^2}{3\beta\tilde{\lambda}^2\gamma(\gamma_1+\gamma_2)} \frac{1}{d\mathcal{F}^2}, \tag{25}$$

where we have expanded the expression for $|\langle \theta_j \rangle_{\text{e}}|^2$ (see Eq. (17) in the Methods section in the article) in the limit of large detuning. $\beta_2 = \frac{|\mu_{g1}|^2 + |\mu_{g2}|^2}{|\mu_g|^2}$ is the ratio between the Clebsch-Gordon coefficients of the transitions characterized by $\tilde{g}_1, \tilde{g}_2$ and g in Supplementary Figure 3b. For the experimental Cs-cells and a detuning of $\Delta = 2\pi \cdot 898$ MHz, we find that $N_{\text{clas}} \sim \frac{7.4 \cdot 10^{11}}{d\mathcal{F}^2}$. With $d = 168$ and $\mathcal{F} = 100$ this gives $N_{\text{clas}} \sim 4.4 \cdot 10^5$. Since the quantum and classical field differ both in polarisation and frequency, this level of filtering is expected to be easily achieved using using a combination of both polarisation filtering and the filter-cavity.

Readout. Here, we give the expressions for the second order correction to the readout efficiency and present

the details of the simulations. For the second order term $\eta_{\text{read},2}$ we find

$$\eta_{\text{read},2} = 2\kappa_1 \text{Real}\Bigg(\int_0^{\tau_{\text{read}}} dt \int_0^{t} dt' \int_0^{t'} dt'' \frac{\sqrt{N}\bar{\mathcal{B}}^*}{2|\mathcal{D}|} e^{\text{Real}(\bar{\mathcal{A}}+\bar{\mathcal{C}})t} \left(e^{\frac{1}{2}\sqrt{\mathcal{D}}^* t} - e^{-\frac{1}{2}\sqrt{\mathcal{D}}^* t} \right) e^{\bar{\mathcal{C}}(t'-t'')} e^{-\frac{1}{2}(\bar{\mathcal{A}}+\bar{\mathcal{C}})(t'-t'')}$$
$$\times \Bigg(e^{\frac{1}{2}\sqrt{\mathcal{D}}t} \Big(e^{\frac{-1}{2}\sqrt{\mathcal{D}}(t'-t'')} - e^{\frac{-1}{2}\sqrt{\mathcal{D}}(t'+t'')} \Big) \Bigg(\Big[\Big(\bar{\mathcal{A}} - \bar{\mathcal{C}} + \sqrt{\mathcal{D}} \Big) N \langle \delta\mathcal{B}_j(t')\delta\mathcal{B}_j(t'')\rangle_{\text{e}} + 2\bar{\mathcal{B}} N \langle \delta\mathcal{C}_j(t')\delta\mathcal{B}_j(t'')\rangle_{\text{e}} \Big] \frac{\sqrt{N}\bar{\mathcal{B}}}{\sqrt{\mathcal{D}}} \Bigg)$$
$$+ e^{\frac{1}{2}\sqrt{\mathcal{D}}(t+t''-t')} \Bigg(\Big[\Big(\bar{\mathcal{A}} - \bar{\mathcal{C}} + \sqrt{\mathcal{D}} \Big) N \langle \delta\mathcal{B}_j(t')\delta\mathcal{C}_j(t'')\rangle_{\text{e}} + 2\bar{\mathcal{B}}\sqrt{N} \langle \delta\mathcal{C}_j(t')\delta\mathcal{C}_j(t'')\rangle_{\text{e}} \Big] \frac{\bar{\mathcal{A}} - \bar{\mathcal{C}} + \sqrt{\mathcal{D}}}{2\sqrt{\mathcal{D}}} \Bigg)$$
$$+ e^{\frac{1}{2}\sqrt{\mathcal{D}}(t-t''-t')} \Bigg(\Big[\Big(\bar{\mathcal{A}} - \bar{\mathcal{C}} + \sqrt{\mathcal{D}} \Big) N \langle \delta\mathcal{B}_j(t')\delta\mathcal{C}_j(t'')\rangle_{\text{e}} + 2\bar{\mathcal{B}}\sqrt{N} \langle \delta\mathcal{C}_j(t')\delta\mathcal{C}_j(t'')\rangle_{\text{e}} \Big] \frac{-\bar{\mathcal{A}} + \bar{\mathcal{C}} + \sqrt{\mathcal{D}}}{2\sqrt{\mathcal{D}}} \Bigg)$$
$$+ e^{-\frac{1}{2}\sqrt{\mathcal{D}}t} \Big(e^{\frac{1}{2}\sqrt{\mathcal{D}}(t'-t'')} - e^{\frac{1}{2}\sqrt{\mathcal{D}}(t'+t'')} \Big) \Bigg(\Big[\Big(\bar{\mathcal{A}} - \bar{\mathcal{C}} - \sqrt{\mathcal{D}} \Big) N \langle \delta\mathcal{B}_j(t')\delta\mathcal{B}_j(t'')\rangle_{\text{e}} + 2\bar{\mathcal{B}} N \langle \delta\mathcal{C}_j(t')\delta\mathcal{B}_j(t'')\rangle_{\text{e}} \Big] \frac{\sqrt{N}\bar{\mathcal{B}}}{\sqrt{\mathcal{D}}} \Bigg)$$
$$+ e^{\frac{1}{2}\sqrt{\mathcal{D}}(t'+t''-t)} \Bigg(\Big[\Big(\bar{\mathcal{A}} - \bar{\mathcal{C}} - \sqrt{\mathcal{D}} \Big) N \langle \delta\mathcal{B}_j(t')\delta\mathcal{C}_j(t'')\rangle_{\text{e}} + 2\bar{\mathcal{B}}\sqrt{N} \langle \delta\mathcal{C}_j(t')\delta\mathcal{C}_j(t'')\rangle_{\text{e}} \Big] \frac{\bar{\mathcal{A}} - \bar{\mathcal{C}} - \sqrt{\mathcal{D}}}{2\sqrt{\mathcal{D}}} \Bigg)$$
$$+ e^{\frac{1}{2}\sqrt{\mathcal{D}}(t'-t''-t)} \Bigg(\Big[\Big(\bar{\mathcal{A}} - \bar{\mathcal{C}} - \sqrt{\mathcal{D}} \Big) N \langle \delta\mathcal{B}_j(t')\delta\mathcal{C}_j(t'')\rangle_{\text{e}} + 2\bar{\mathcal{B}}\sqrt{N} \langle \delta\mathcal{C}_j(t')\delta\mathcal{C}_j(t'')\rangle_{\text{e}} \Big] \frac{-\bar{\mathcal{A}} + \bar{\mathcal{C}} - \sqrt{\mathcal{D}}}{2\sqrt{\mathcal{D}}} \Bigg) \Bigg) \Bigg). \quad (26)$$

Here we have neglected the contributions from the fluctuations contained in $\delta\mathcal{A}(t)$ and $\mathcal{B}_0$ since they are suppressed by a factor of at least $d\mathcal{F}/N$ compared to the terms above. In deriving Eq. (26), we have used that $\hat{a}_{\text{cell}}^{(2)}$ consists of sums of the form

$$\frac{1}{N} \sum_{l=1}^{N-1} \sum_{j=1}^{N} \sum_{j'=1}^{N} e^{-2i\pi/N(j-j')l} \delta X_j(t')\delta X_{j'}(t''), \quad (27)$$

where X_j could e.g. denote $\mathcal{B}_j$. For $\eta_{\text{read},2}$, we calculate $\langle \hat{a}_{\text{cell}}^{(0)} \hat{a}_{\text{cell}}^{(2)} \rangle$ and the average of Eq. (27) is approximatively $N\langle \delta X_j(t')\delta X_j(t'')\rangle_{\text{e}}$ because $\langle \delta X_j(t')\delta X_{j'}(t'')\rangle_{\text{e}} = 0$, if $j \neq j'$, since the motion of different atoms are uncorrelated and we have assumed that $N-1 \approx N$. All correlations appearing in Eq. (26) are thus single atom correlations and the index j is kept to indicate this. The correlations contained in $\eta_{\text{read},2}$ can be treated analytically, in a similar fashion as the correlations in $\langle |\theta_j(t)|^2 \rangle_{\text{e}}$ for the write process, but we have instead simulated the correlations numerically for the previously mentioned Cs-cells.

Numerical simulation - Read. The simulations are performed in the same way as for the write process. An extra difficulty is, however, that we consider the coupling between the light fields and the extra levels in ^{133}Cs. We assume that the readout process has the level structure shown in Supplementary Figure 4.

The couplings to the extra levels result in extra coupling terms in the expressions for $\mathcal{A}, \mathcal{B}_j$ and $\mathcal{C}_j$, which we include, but the expression for $\eta_{\text{read},2}$ is still the same as given in Eq. (26). Note, however, that a cavity detuning of the quantum field (appearing in the expression for $\mathcal{A}$) on the order of κ_1 is needed to compensate the phases resulting from some of these additional couplings. The starting point of our numerical simulations is therefore Eq. (26), where we can change the order of integration and introduce the variables $u = t' + t''$ and $s = t' - t''$ since the correlations only depend on the time difference $|t' - t''|$. Performing the integrals over t and u analytically, allows us to write

$$\eta_{\text{read},2} = \int_0^{\tau_{\text{read}}} (h_1(\tau_{\text{read}}, s)\langle \delta\mathcal{B}, \delta\mathcal{B}\rangle_{\text{e}}(s) + h_2(\tau_{\text{read}}, s)\langle \delta\mathcal{B}, \delta\mathcal{C}\rangle_{\text{e}}(s)$$
$$+ h_3(\tau_{\text{read}}, s)\langle \delta\mathcal{C}, \delta\mathcal{B}\rangle_{\text{e}}(s) + h_4(\tau_{\text{read}}, s)\langle \delta\mathcal{C}, \delta\mathcal{C}\rangle_{\text{e}}(s))ds, \quad (28)$$

where $h_1(\tau_{\text{read}}, s), h_2(\tau_{\text{read}}, s), h_3(\tau_{\text{read}}, s)$ and $h_4(\tau_{\text{read}}, s)$ are functions of s and τ_{read}, which are obtained from the integration over t and u. We have once again introduced the short notation for the correlations $\langle \delta\mathcal{B}_j(t')\delta\mathcal{C}_j(t'')\rangle_{\text{e}} = \langle \delta\mathcal{B}, \delta\mathcal{C}\rangle_{\text{e}}(s)$. Note that $\langle \delta\mathcal{B}, \delta\mathcal{C}\rangle_{\text{e}}(s) \to 0$ for $s \to \infty$ similar to the situation in the write process, i.e., the coupling of an atom at time t is uncorrelated from its initial coupling if t is large. We can therefore introduce a cutoff $s_{\max}$ in the integral in Eq. (28) such that we can evaluate $\eta_{\text{read},2}$ for an arbitrary length of the readout pulse τ_{read} without additional numerical difficulty. We then numerically evaluate $\eta_{\text{read},2}$ from Eq. (28) by simulating

the decay of the correlations similar to the simulation of the write process. From the numerical simulation, we find that the term $\langle \delta\mathcal{C}_j \delta\mathcal{C}_j \rangle_e$ dominates $\eta_{\text{read},2}$. This term describes loss of the excitation due to spontaneous emission to modes not confined by the cavity.

Errors. Here, we give the detailed expression for the probability (p_1) to read out incoherent photons to first order. The incoherent photons are being readout from assymetric modes described by the operators

$$\hat{S}_l = \frac{1}{\sqrt{N}} \sum_{j=1}^{N} e^{2i\pi(j-1)l/N} \hat{\sigma}_{01}^{(j)}, \tag{29}$$

where $l = \{1, 2, \ldots, N-1\}$. Note that the symmetric Dicke mode is described by $\hat{S}_0$. From the pertubative expansion of $\hat{a}_{\text{cell}}$, we find that the assymetric modes gives a first order contribution of

$$\begin{aligned}\hat{a}_{\text{cell}}^{(1)}(t) = \sqrt{\epsilon} \int_0^t dt' \frac{e^{\frac{1}{2}(\bar{\mathcal{A}}+\bar{\mathcal{C}})(t-t')}}{2\sqrt{\mathcal{D}}} e^{\bar{\mathcal{C}}t'} \frac{1}{\sqrt{N}} \sum_{l=1}^{N-1} \sum_{j=1}^{N} e^{-2i\pi(j-1)l/N} \Big(\\ \left(e^{\frac{1}{2}\sqrt{\mathcal{D}}(t-t')} - e^{-\frac{1}{2}\sqrt{\mathcal{D}}(t-t')}\right) \left(\left(\bar{\mathcal{A}} - \bar{\mathcal{C}}\right) \delta\mathcal{B}_j(t') + 2\bar{\mathcal{B}}\delta\mathcal{C}_j(t')\right) \hat{S}_l \\ + \left(e^{\frac{1}{2}\sqrt{\mathcal{D}}(t-t')} + e^{-\frac{1}{2}\sqrt{\mathcal{D}}(t-t')}\right) \sqrt{\mathcal{D}}\delta\mathcal{B}_j(t')\hat{S}_l \Big).\end{aligned} \tag{30}$$

The probability to read out the incoherent photons is then

$$p_1 = \frac{\kappa_2^2 \kappa_1}{4} \int_0^{\tau_{read}} dt \int_0^t dt' \int_0^t dt'' e^{-\kappa_2/2(2t-t'-t'')} \langle (\hat{a}_{\text{cell}}^{(1)}(t'))^\dagger \hat{a}_{\text{cell}}^{(1)}(t'') \rangle. \tag{31}$$

We can numerically evaluate the correlations contained in Eq. (31) in a similar fashion as the correlations in $\eta_{\text{read},2}$, i.e., we simulate the experimental Cs-cells. Thereby, we get the results presented in Fig. 3b in the article.

DLCZ error analysis. In order to fully characterize the performance of a DLCZ repeater based on the room temperature cells, we investigate how the errors from incoherent photons propagate in the repeater. First, we consider the state being produced in the entanglement generation step (see above). We define the following parameters:

- η_{write} : The write efficiency, which basically is the probability to have a Dicke state in the ensemble conditioned on a quantum photon being emitted. If we are not in the right state we are dominated by the intitial state and thus with probability $1 - \eta_{\text{write}}$, we assume the atomic state to be $|00\ldots0\rangle$.
- p_e : The excitation probability, which depends on the driving strength.
- P_{d} : The dark count probability of a detector. We estimate this as $P_{\text{d}} \sim r_{dark} t_{\text{int}}$ where r_{dark} is the dark count rate and t_{int} is the length of the driving pulse.
- η_{d} : The detection efficiency, which is determined by the total efficiency of the detector, the outcoupling losses and the transmission losses from the cavity to the detector.

To second order in the excitation probability, the state of the two ensembles following a single click in a detector at the central station is described by the density matrix

$$\begin{aligned}\rho_{\text{success}} = \Bigg[& \Big[p_e^2(1-\eta_{\text{write}})^2(2\eta_{\text{d}} - 2\eta_{\text{d}}^2)(1-P_{\text{d}})^2 + 2p_e^2(1-\eta_{\text{write}})^2(1-\eta_{\text{d}})^2 P_{\text{d}}(1-P_{\text{d}}) \\ & + 2(1-p_e)^2 P_{\text{d}}(1-P_{\text{d}}) + 4p_e(1-\eta_{\text{write}})(1-p_e)(1-\eta_{\text{d}})P_{\text{d}}(1-P_{\text{d}}) \\ & + 4p_e^2(1-\eta_{\text{write}})^2(\eta_{\text{d}} - \eta_{\text{d}}^2)(1-P_{\text{d}})^2 + 4p_e^2(1-\eta_{\text{write}})^2(1-\eta_{\text{d}})^2 P_{\text{d}}(1-P_{\text{d}})\Big] |\mathbf{00}\rangle\langle\mathbf{00}| \\ & + \Big[p_e^2\eta_{\text{write}}^2(2\eta_{\text{d}} - 2\eta_{\text{d}}^2)(1-P_{\text{d}})^2 + 2p_e^2\eta_{\text{write}}^2(1-\eta_{\text{d}})^2 P_{\text{d}}(1-P_{\text{d}})\Big] |\mathbf{11}\rangle\langle\mathbf{11}| \\ & + \Big[p_e^2\eta_{\text{write}}(1-\eta_{\text{write}})(2\eta_{\text{d}} - 2\eta_{\text{d}}^2)(1-P_{\text{d}})^2 + 2p_e^2\eta_{\text{write}}(1-\eta_{\text{write}})(1-\eta_{\text{d}})^2 P_{\text{d}}(1-P_{\text{d}}) \\ & + 2p_e\eta_{\text{write}}(1-p_e)(1-\eta_{\text{d}})P_{\text{d}}(1-P_{\text{d}}) + 4p_e^2\eta_{\text{write}}(1-\eta_{\text{write}})(\eta_{\text{d}} - \eta_{\text{d}}^2)(1-P_{\text{d}})^2\end{aligned}$$

$$+4p_e^2\eta_{\rm write}(1-\eta_{\rm write})(1-\eta_{\rm d})^2P_{\rm d}(1-P_{\rm d})\Big](|\mathbf{01}\rangle\langle\mathbf{01}|+|\mathbf{10}\rangle\langle\mathbf{10}|)$$
$$+\Big[2p_e^2\eta_{\rm write}^2(\eta_{\rm d}-\eta_{\rm d}^2)(1-P_{\rm d})^2+2p_e^2\eta_{\rm write}^2(1-\eta_{\rm d})^2P_{\rm d}(1-P_{\rm d})\Big](|\mathbf{20}\rangle\langle\mathbf{20}|+|\mathbf{02}\rangle\langle\mathbf{02}|)$$
$$+2p_e\eta_{\rm write}(1-p_e)\eta_{\rm d}(1-P_{\rm d})^2|\mathbf{\Psi}\rangle\langle\mathbf{\Psi}|\Bigg]\frac{1}{\mathcal{N}}, \quad (32)$$

where we have defined $|\mathbf{0}\rangle=|00\ldots0\rangle$, $|\mathbf{1}\rangle=|\text{Dicke}\rangle$, $|\mathbf{2}\rangle=\frac{1}{\sqrt{N}}\sum_{i,j=1}^{N}|1\rangle_i|1\rangle_j\langle0|_i\langle0|_j|00\ldots0\rangle$ and $|\mathbf{\Psi}\rangle=\frac{1}{\sqrt{2}}(|\mathbf{01}\rangle+|\mathbf{10}\rangle)$. $\mathcal{N}=P_{\rm success}$ is a normalization constant, which gives the success probability of the operation. Note that we have assumed number resolving detectors. It is seen from Eq. (32) that we can write $\rho_{\rm success}=a_0|\mathbf{\Psi}\rangle\langle\mathbf{\Psi}|+b_0|\mathbf{00}\rangle\langle\mathbf{00}|+c_0(|\mathbf{01}\rangle\langle\mathbf{01}|+|\mathbf{10}\rangle\langle\mathbf{10}|)+d_0|\mathbf{11}\rangle\langle\mathbf{11}|+e_0(|\mathbf{02}\rangle\langle\mathbf{02}|+|\mathbf{20}\rangle\langle\mathbf{20}|)$.

We now consider the entanglement swapping of two states of the form $\rho_{\rm success}$ using the setup shown in Supplementary Figure 5. In the swap, an ensemble from each entangled pair is read out and the corresponding photons are combined on a balanced beam splitter. With probability a_0^2, we are swapping two states of the form $|\mathbf{\Psi}\rangle$ and the state after a successful swap is

$$\rho_{a_0^2}=\Bigg[\frac{1}{4}\Big[2\eta_{\rm dr}(1-\eta_{\rm dr})(1-P_{\rm dr})^2+2(1-\eta_{\rm dr})^2P_{\rm dr}(1-P_{\rm dr})\Big]|\mathbf{00}\rangle\langle\mathbf{00}|$$
$$+\frac{1}{4}\Big[2P_{\rm dr}(1-P_{\rm dr})\Big]|\mathbf{11}\rangle\langle\mathbf{11}|$$
$$+\frac{1}{4}\Big[2(1-\eta_{\rm dr})P_{\rm dr}(1-P_{\rm dr})\Big](|\mathbf{01}\rangle\langle\mathbf{01}|+|\mathbf{10}\rangle\langle\mathbf{10}|)$$
$$+\frac{1}{2}\eta_{\rm dr}(1-P_{\rm dr})^2|\Psi\rangle\langle\Psi|\Bigg]\frac{1}{\mathcal{N}'}, \quad (33)$$

where we have introduced the total readout detection efficiency $\eta_{\rm dr}$, which is determined by the readout efficiency, the outcoupling losses and the efficiency of the detectors. In contrast to the detection efficiency in the entanglement generation, the readout detection efficiency does not include fiber losses since the entanglement swap is a local process. $\mathcal{N}'$ is a normalization constant. Furthermore, we have defined the readout dark count rate $P_{\rm dr}$, which contains the probability of reading out incoherent photons from the ensembles and the detector dark counts, i.e. $P_{\rm dr}=r_{\rm dark}\tau_{\rm read}+\eta_{\rm dr}p_1$. Here $r_{\rm dark}\tau_{\rm read}$ is the dark count rate of the detectors and p_1 is the probability to emit incoherent photons. As previously described, p_1 is mainly determined by the inefficiency of the optical pumping in the initialization of the ensembles and the memory time of the ensemble. We will neglect the limited memory time and simply assume a fixed value of p_1 from the inefficiency of the optical pumping. From Eq. (33), we can express $\rho_{a_0^2}$ as

$$\rho_{a_0^2}=\frac{1}{\mathcal{N}'}\Bigg[\frac{1}{4}\alpha|\mathbf{00}\rangle\langle\mathbf{00}|+\frac{1}{4}(\beta-\eta_{\rm dr}(1-P_{\rm dr})^2)(|\mathbf{01}\rangle\langle\mathbf{01}|+|\mathbf{10}\rangle\langle\mathbf{10}|)$$
$$+\frac{1}{4}\lambda|\mathbf{11}\rangle\langle\mathbf{11}|+\frac{1}{2}\eta_{\rm dr}(1-P_{\rm dr})^2|\mathbf{\Psi}\rangle\langle\mathbf{\Psi}|\Bigg], \quad (34)$$

with α,β, and λ given by Eq. (33). Considering all the combinations from swapping two states of the form $\rho_{\rm success}$, we find that the output state can be written as

$$\rho_{\rm swap,1}=\frac{1}{\mathcal{N}''}\Bigg[\Big[\frac{a_0^2}{4}\alpha+b_0^2\lambda+c_0^2\alpha+b_0a_0\beta+c_0a_0\alpha+2b_0c_0\beta+2b_0e_0\tilde{\beta}+2c_0e_0\tilde{\alpha}+e_0^2\tilde{\gamma}+a_0e_0\tilde{\alpha}\Big]|\mathbf{00}\rangle\langle\mathbf{00}|$$
$$+\Big[\frac{a_0^2}{4}(\beta-\eta_{\rm dr}(1-P_{\rm dr})^2)+\frac{a_0b_0}{2}\lambda+a_0c_0\beta+\frac{a_0d_0}{2}\alpha+b_0c_0\lambda+b_0d_0\beta+c_0^2\beta+c_0d_0\alpha$$
$$+\frac{a_0e_0}{2}\tilde{\beta}+d_0e_0\tilde{\alpha}\Big](|\mathbf{01}\rangle\langle\mathbf{01}|+|\mathbf{10}\rangle\langle\mathbf{10}|)$$
$$+\Big[\frac{a_0^2}{4}\lambda+c_0^2\lambda+d_0^2\alpha+c_0a_0\lambda+d_0a_0\beta+2c_0d_0\beta\Big]|\mathbf{11}\rangle\langle\mathbf{11}|$$
$$+\Big[\frac{a_0e_0}{2}\beta+b_0e_0\lambda+c_0e_0\beta+2e_0^2\tilde{\beta}\Big](|\mathbf{02}\rangle\langle\mathbf{02}|+|\mathbf{20}\rangle\langle\mathbf{20}|)$$

$$+\Big[\frac{a_0e_0}{2}\lambda+c_0e_0\lambda+d_0e_0\beta\Big](|\mathbf{12}\rangle\langle\mathbf{12}|+|\mathbf{21}\rangle\langle\mathbf{21}|)$$
$$+e_0^2\lambda|\mathbf{22}\rangle\langle\mathbf{22}|$$
$$+\frac{a_0^2}{2}\eta_{\rm dr}(1-P_{\rm dr})|\mathbf{\Psi}\rangle\langle\mathbf{\Psi}|\Bigg], \tag{35}$$

where we have defined

$$\tilde{\alpha}=3\eta_{\rm dr}(1-\eta_{\rm dr})^2(1-P_{\rm dr})^2+2(1-\eta_{\rm dr})^3P_{\rm dr}(1-P_{\rm dr}) \tag{36}$$
$$\tilde{\beta}=2\eta_{\rm dr}(1-\eta_{\rm dr})(1-P_{\rm dr})^2+2(1-\eta_{\rm dr})^2P_{\rm dr}(1-P_{\rm dr}) \tag{37}$$
$$\tilde{\gamma}=4\eta_{\rm dr}(1-\eta_{\rm dr})^3(1-P_{\rm dr})^2+2(1-\eta_{\rm dr})^4P_{\rm dr}(1-P_{\rm dr}), \tag{38}$$

and $\mathcal{N}''=P_{\rm swap,1}$ is a normalization constant, which gives the success probability of the swap operation. It is seen that we can write

$$\rho_{\rm swap,1}=a_1|\mathbf{\Psi}\rangle\langle\mathbf{\Psi}|+b_1|\mathbf{00}\rangle\langle\mathbf{00}|+c_1(|\mathbf{01}\rangle\langle\mathbf{01}|+|\mathbf{10}\rangle\langle\mathbf{10}|)+d_1|\mathbf{11}\rangle\langle\mathbf{11}|+e_1(|\mathbf{02}\rangle\langle\mathbf{02}|+|\mathbf{20}\rangle\langle\mathbf{20}|)$$
$$+f_1(|\mathbf{12}\rangle\langle\mathbf{12}|+|\mathbf{21}\rangle\langle\mathbf{21}|)+g_1|\mathbf{22}\rangle\langle\mathbf{22}| \tag{39}$$

with constants a_1,b_1,c_1,d_1,e_1,f_1 and g_1 determined by Eq. (35).

The vacuum part of the swapped state grows exponentially with the number of swaps in the DLCZ protocol [6] and it is therefore necessary to perform a final postselection where two entangled states are combined (see Supplementary Figure 5).

We assume two parties named Alice and Bob who share two entangled pairs such that Alice has ensemble 1 and 3 and Bob has ensemble 2 and 4. Ensemble 1 and 2 are entangled and so is ensemble 3 and 4. The intial state is then

$$\rho_{\rm intial}=\Big(a|\mathbf{\Psi}\rangle\langle\mathbf{\Psi}|+b|\mathbf{00}\rangle\langle\mathbf{00}|+c(|\mathbf{01}\rangle\langle\mathbf{01}|+|\mathbf{10}\rangle\langle\mathbf{10}|)+d|\mathbf{11}\rangle\langle\mathbf{11}|$$
$$+e(|\mathbf{02}\rangle\langle\mathbf{02}|+|\mathbf{20}\rangle\langle\mathbf{20}|)+f(|\mathbf{12}\rangle\langle\mathbf{12}|+|\mathbf{21}\rangle\langle\mathbf{21}|)+g|\mathbf{22}\rangle\langle\mathbf{22}|\Big)_{1,2}$$
$$\otimes\Big(a|\mathbf{\Psi}\rangle\langle\mathbf{\Psi}|+b|\mathbf{00}\rangle\langle\mathbf{00}|+c(|\mathbf{01}\rangle\langle\mathbf{01}|+|\mathbf{10}\rangle\langle\mathbf{10}|)+d|\mathbf{11}\rangle\langle\mathbf{11}|$$
$$+e(|\mathbf{02}\rangle\langle\mathbf{02}|+|\mathbf{20}\rangle\langle\mathbf{20}|)+f(|\mathbf{12}\rangle\langle\mathbf{12}|+|\mathbf{21}\rangle\langle\mathbf{21}|)+g|\mathbf{22}\rangle\langle\mathbf{22}|\Big)_{3,4} \tag{40}$$

All ensembles are now readout in, e.g., a cryptography scheme and a success is conditioned on both Alice and Bob recording a single click. The total success probability is

$$P_{\rm ps}=\frac{a^2}{2}(\alpha_{\rm ps}\beta_{\rm ps}+\gamma_{\rm ps}^2)+b^2\alpha_{\rm ps}^2+2c^2\alpha_{\rm ps}\beta_{\rm ps}+d^2\beta_{\rm ps}^2+2c^2\gamma_{\rm ps}^2+2ab\alpha_{\rm ps}\gamma_{\rm ps}$$
$$+2ac(\alpha_{\rm ps}\beta_{\rm ps}+\gamma_{\rm ps}^2)+2ad\beta_{\rm ps}\gamma_{\rm ps}+4bc\alpha_{\rm ps}\gamma_{\rm ps}+4cd\beta_{\rm ps}\gamma_{\rm ps}+2bd\gamma_{\rm ps}^2$$
$$+2ae(\alpha_{\rm ps}\tilde{\beta}_{\rm ps}+\gamma_{\rm ps}\beta_{\rm ps})+2af(\gamma_{\rm ps}\tilde{\beta}_{\rm ps}+\beta_{\rm ps}^2)+2ag\beta_{\rm ps}\tilde{\beta}_{\rm ps}+4be\alpha_{\rm ps}\beta_{\rm ps}$$
$$+4bf\gamma_{\rm ps}\beta_{\rm ps}+2bg\beta_{\rm ps}^2+4ce(\gamma_{\rm ps}\beta_{\rm ps}+\alpha_{\rm ps}\tilde{\beta}_{\rm ps})+4cf(\beta_{\rm ps}^2+\gamma_{\rm ps}\tilde{\beta}_{\rm ps})+4cg\beta_{\rm ps}\tilde{\beta}_{\rm ps}+4de\gamma_{\rm ps}\tilde{\beta}_{\rm ps}$$
$$+4df\beta_{\rm ps}\tilde{\beta}_{\rm ps}+2dg\tilde{\beta}_{\rm ps}^2+2e^2(\alpha_{\rm ps}\tilde{\gamma}_{\rm ps}+\beta_{\rm ps}^2)+4eg\beta_{\rm ps}\tilde{\gamma}_{\rm ps}+4fg\tilde{\beta}_{\rm ps}\tilde{\gamma}_{\rm ps}+g^2\tilde{\gamma}_{\rm ps}^2$$
$$+4fe(\gamma_{\rm ps}\tilde{\gamma}_{\rm ps}+\tilde{\beta}_{\rm ps}\beta_{\rm ps})+2f^2(\tilde{\gamma}_{\rm ps}\beta_{\rm ps}+\tilde{\beta}^2), \tag{41}$$

where we have defined

$$\alpha_{\rm ps}=2P_{\rm dr}(1-P_{\rm dr}) \tag{42}$$
$$\beta_{\rm ps}=2\eta_{\rm dr}(1-\eta_{\rm dr})(1-P_{\rm dr})^2+2(1-\eta_{\rm dr})^2P_{\rm dr}(1-P_{\rm dr}) \tag{43}$$
$$\gamma_{\rm ps}=\eta_{\rm dr}(1-P_{\rm dr})^2+2(1-\eta_{\rm dr})P_{\rm dr}(1-P_{\rm dr}) \tag{44}$$
$$\tilde{\beta}_{\rm ps}=3\eta_{\rm dr}(1-\eta_{\rm dr})^2(1-P_{\rm dr})^2+2(1-\eta_{\rm dr})^3P_{\rm dr}(1-P_{\rm dr}) \tag{45}$$
$$\tilde{\gamma}_{\rm ps}=4\eta_{\rm dr}(1-\eta_{\rm dr})^3(1-P_{\rm dr})^2+2(1-\eta_{\rm dr})^4P_{\rm dr}(1-P_{\rm dr}). \tag{46}$$

The postselected fidelity of the state is

$$F_{\rm ps}=\frac{\frac{a^2}{4}\eta_{\rm read}^2\eta_{\rm dr}^2(1-P_{\rm dr})^4(1+\cos(\phi-\theta))+\frac{a^2}{4}(\gamma_{\rm ps}^2-\eta_{\rm read}^2\eta_{\rm dr}^2(1-P_{\rm dr})^4)+c^2\gamma_{\rm ps}^2+ac\gamma_{\rm ps}^2}{P_{\rm ps}}, \tag{47}$$

where the phases ϕ, θ can be different due to variations in the path lengths of the photons being read out. In the ideal case, we have $\phi = \theta$ for which the fidelity is maximal.

The rate of a DLCZ repeater based on the room temperature cells can be estimated as [6]

$$r \approx \left(\frac{2}{3}\right)^{n+1} P_0 P_{\text{swap},1} P_{\text{swap},2} \ldots P_{\text{swap},n} P_{\text{ps}} \frac{2^n c}{L} \tag{48}$$

where n is the number of swap levels in the repeater and L is the distance. Note, however, that the scalability of the room temperature cells enables spatially multiplexing, which both increases the rate of the repeater and decreases the necessary memory time of the ensembles [6]. Assuming that $2M$ ensembles are used at each repeater station, the rate will increase by a factor of M. We have considered a basic repeater segment consisting of only a single swap without multiplexing in order to estimate the rate and fidelity of a distributed pair. We assume that the distance to distribute entanglement over is $L = 80$ km and that the losses in the fibers are given by the attenuation length at telecom wavelengths, which is ~20 km. Furthermore, we assume SPD efficiencies of 95% and dark count rates of 1 Hz. This reflects what is possible with current superconducting detectors [7, 8]. We then perform an optimization in all the parameters characterizing the cells, e.g. the excitation probability, the write time and the readout time. We include experimental imperfections such as outcoupling losses of around 10% and intracavity losses of 2%. As a result we find that a pair with fidelity $\sim 80\%$ can be distributed with a rate of ~ 0.2 Hz. We have assumed that $\epsilon \approx 0.5\%$ and have neglected effects from finite memory time of the atoms. Furthermore, we have assumed that $\phi = \theta$ (see Supplementary Figure 5).

Supplementary References

[1] A. V. Gorshkov, A. André, M. D. Lukin, and A. S. Sørensen, “Photon storage in Λ-type optically dense atomic media. i. cavity model,” *Phys. Rev. A*, vol. 76, p. 033804, 2007.

[2] A. Gorshkov, A. André, M. Lukin, and A. Sørensen, “Photon storage in Λ-type optically dense atomic media. iii. effects of inhomogeneous broadening,” *Phys. Rev. A*, vol. 76, p. 033806, 2007.

[3] J. D. Jackson, *Classical Electrodynamics*. John Wiley and Sons, Inc., 1975.

[4] K. Hammerer, A. S. Sørensen, and E. S. Polzik, “Quantum interface between light and atomic ensembles,” *Rev. Mod. Phys.*, vol. 82, pp. 1041–1093, 2010.

[5] K. Jensen, *Quantum Information, Entanglement and Magnetometry with macroscopic Gas Samples and Non-Classical Light*. PhD thesis, The Niels Bohr Institute, University of Copenhagen, February 2011.

[6] N. Sangouard, C. Simon, H. de Riedmatten, and N. Gisin, “Quantum repeaters based on atomic ensembles and linear optics,” *Rev. Mod. Phys.*, vol. 83, pp. 33–80, 2011.

[7] A. E. Lita, A. J. Miller, and S. W. Nam, “Counting near-infrared single photons with 95% efficiency,” *Optics Express*, vol. 16, no. 5, pp. 3032–3040, 2008.

[8] D. H. Smith, G. Gillett, M. P. de Almeida, C. Branciard, A. Fedrizzi, T. J. Weinhold, A. Lita, B. Calkins, T. Gerrits, H. M. Wiseman, S. W. Nam, and A. G. White, “Conclusive quantum steering with superconducting transition-edge sensors,” *Nature Communications*, vol. 3, p. 625, 2012.
